# Agent-Based Modeling of Mitochondria Links Sub-Cellular Dynamics to Cellular Homeostasis and Heterogeneity

**DOI:** 10.1371/journal.pone.0168198

**Published:** 2017-01-06

**Authors:** Giovanni Dalmasso, Paula Andrea Marin Zapata, Nathan Ryan Brady, Anne Hamacher-Brady

**Affiliations:** 1 Lysosomal Systems Biology, German Cancer Research Center (DKFZ) and BioQuant, University of Heidelberg, Heidelberg, Germany; 2 Systems Biology of Cell Death Mechanisms, German Cancer Research Center (DKFZ) and BioQuant, University of Heidelberg, Heidelberg, Germany; 3 W. Harry Feinstone Department of Molecular Microbiology & Immunology, Johns Hopkins University Bloomberg School of Public Health, Baltimore, Maryland, United States of America; National University of Singapore, SINGAPORE

## Abstract

Mitochondria are semi-autonomous organelles that supply energy for cellular biochemistry through oxidative phosphorylation. Within a cell, hundreds of mobile mitochondria undergo fusion and fission events to form a dynamic network. These morphological and mobility dynamics are essential for maintaining mitochondrial functional homeostasis, and alterations both impact and reflect cellular stress states. Mitochondrial homeostasis is further dependent on production (biogenesis) and the removal of damaged mitochondria by selective autophagy (mitophagy). While mitochondrial function, dynamics, biogenesis and mitophagy are highly-integrated processes, it is not fully understood how systemic control in the cell is established to maintain homeostasis, or respond to bioenergetic demands. Here we used agent-based modeling (ABM) to integrate molecular and imaging knowledge sets, and simulate population dynamics of mitochondria and their response to environmental energy demand. Using high-dimensional parameter searches we integrated experimentally-measured rates of mitochondrial biogenesis and mitophagy, and using sensitivity analysis we identified parameter influences on population homeostasis. By studying the dynamics of cellular subpopulations with distinct mitochondrial masses, our approach uncovered system properties of mitochondrial populations: (1) mitochondrial fusion and fission activities rapidly establish mitochondrial sub-population homeostasis, and total cellular levels of mitochondria alter fusion and fission activities and subpopulation distributions; (2) restricting the directionality of mitochondrial mobility does not alter morphology subpopulation distributions, but increases network transmission dynamics; and (3) maintaining mitochondrial mass homeostasis and responding to bioenergetic stress requires the integration of mitochondrial dynamics with the cellular bioenergetic state. Finally, (4) our model suggests sources of, and stress conditions amplifying, cell-to-cell variability of mitochondrial morphology and energetic stress states. Overall, our modeling approach integrates biochemical and imaging knowledge, and presents a novel open-modeling approach to investigate how spatial and temporal mitochondrial dynamics contribute to functional homeostasis, and how subcellular organelle heterogeneity contributes to the emergence of cell heterogeneity.

## Introduction

Mitochondria are essential sources of ATP, and their morphology is dynamic; mitochondria are highly mobile within a cell [1, 2] and undergo fusion and fission events, resulting in a continuum of morphologies among populations of mitochondria, from tubular to small puncta [3]. Furthermore, mitochondrial homeostasis is dependent on biogenesis through fission-dependent duplication [4] and mitochondrial quality control is carried out by autophagy-mediated degradation, i.e. mitophagy [5–8]. Systems biology studies on mitochondrial morphology have contributed insights into how dynamic mitochondrial behavior relates to homeostasis and functional maintenance. Frequency of fusion and fission cycles determines efficiency of mitophagy [9], and suggests that altered cycles in aging organisms may contribute to maintaining mitochondrial mass [10]. Simulations also suggest that spatial limitations, which decrease fusion and fission capacities during aging, can increase the heterogeneity of mitochondrial genotypes within a cell, and consequently increase heterogeneity among a population of cells [11]. Furthermore, mitochondrial mobility has been predicted to have a role in maintaining a healthy mitochondrial population [12]. Of note, these studies did not address how morphological states and mass homeostasis coordinate bioenergetic supply and demand. Indeed, fission, fusion, biogenesis and mitophagy activities both respond to, and shape, the cellular bioenergetic state [13–20]. Thus, in this study, we sought to analyze how reactions of individual mitochondria form a collective population response, to organize morphological states and maintain mass homeostasis, under basal conditions and in response to bioenergetic stress.

To that end, we employed agent-based modeling (ABM), a computational method to simulate spatial and temporal population activities [21–25], which has been applied in several areas of systems biology, including, apoptotic death receptor dynamics [26], autophagy dynamics [27], dosage screening of drug combinations [28] and lipid composition [29]. This discrete modeling approach involves the repetitive update of rules describing the behavior of autonomous agents, thus relying on computational power to simulate global behavior emerging from collective action of all agents. Using ABM, we simulated individual spatial and temporal behaviors of mitochondria, and bi-directional mitochondria-environment bioenergetic signaling. We analyzed the temporal behavior of mitochondrial subpopulations with variable masses, and report that the distribution of mitochondrial subpopulations is determined by fusion-fission activities and the total mitochondrial mass, but is minimally impacted by the directionality of mitochondrial movement. Further, the time required to establish sub-population homeostasis, and to effectively transmit information among the population, is determined by fusion/fission events and mitochondrial velocities. Our simulations also suggest that the ability of mitochondrial populations to re-establish homeostasis following damage requires environmental bioenergetic stress sensing. Overall, these findings demonstrate that ABM permits a precise investigation of the link between subcellular and cell decisions, and provides a framework for understanding the bidirectional relationship of organelle dynamics with cellular environmental signaling processes, and the emergence of cell heterogeneity.

## Results

### Agent based model of mitochondrial fusion-fission cycles

**Fig 1:**
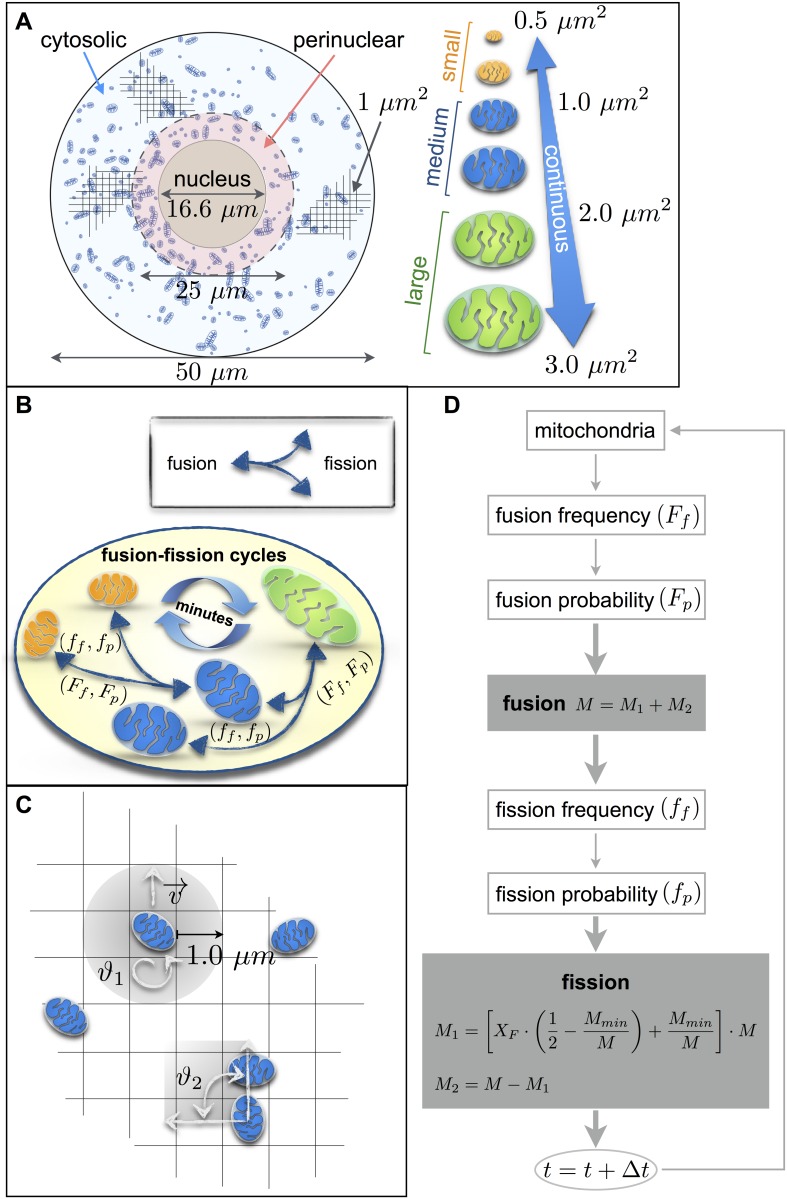
An agent-based model of mitochondrial dynamics determined by organelle mobility and fusion-fission cycles. (A) Model representation. The model consists of a two-dimensional grid (elements of 1 μm × 1 μm) in which the cell is represented by a circle with a diameter of 50 μm, and a nucleus with a diameter of 16.6 μm. The dashed circle (diameter, 25 μm) divides the cell in two regions, perinuclear (red arrow) and cytosolic (blue arrow), with different mitochondrial mobility. The mitochondrial population consists of agents with masses ranging continuously from a minimum value M_min_ of 0.5 μm^2^ to a maximum value Mmax of 3 μm^2^. The mitochondria population is in turn subdivided into three groups: small (mass smaller or equal 1 μm^2^, orange), medium (mass between 1 μm^2^ and 2 μm^2^, blue) and large (mass greater than 2 μm^2^, green). (B) Schematic describing mitochondrial dynamics, consisting of fusion (*f*) and fission (*F*) cycles occurring with temporal frequencies (f_f_, F_f_) of minutes. Mitochondria of all masses are able to undergo fusion and fission events according to fusion and fission probabilities (f_p_, F_p_). Time step used in all the simulation, Δt = 1sec. (C) Schematic describing mitochondrial movement and unfeasible actions. Each mitochondrion checks, with an internal control, the surrounding area with a radius of 1.0 μm in order to avoid unfeasible actions such as moving outside the cell or inside the nucleus and overlapping other mitochondria. If the area is free, the mitochondrion first move one step forward, with a velocity *v*, and than rotates by a random angle ϑ_1_ ϵ [0°, 360°]. If the mitochondrion encounters either another mitochondrion (and no fusion occur), the nucleus, or the cell border, the mitochondrion rotates by a random angle ϑ_2_ ϵ[0°, 90°]. (D) Basic flow chart of the model representing the fusion-fission cycle and main components of the model. Shaded boxes indicate the main processes of the model (fusion and fission) with the respective governing equations.

Mitochondrial fusion and fission processes were modeled using a discrete approach, since a continuous approximation was unreasonable due to the low copy number of mitochondria. To this end, we employed agent-based modeling (ABM), considering mitochondria as discrete agents which can move, fuse with each other, or undergo fission (Fig 1A and 1B). Without explicitly deriving phenomenological equations, time-course ABM simulations proceed by increasing time (t) with a discrete step (Δt = 1s), followed by iterative evaluation of a set of behavioral rules for all mitochondrial agents. Simulations were performed in the NetLogo modeling environment [30] as described in ‘Materials and Methods’. The cellular spatial domain and the set of behavioral rules governing mitochondrial movement, and fusion and fission processes were implemented as follows.

#### Spatial domain and agent’s properties

For simplicity, the cellular domain was modeled in two dimensions, represented as a circle with a diameter of 50 μm containing a central nucleus with a diameter of 16.6 μm [31] and a variable number of mitochondria (Fig 1A). Each mitochondrial agent has the following three properties: mass (M) with units μm^2^, velocity magnitude (v) with units μms^-1^, and directionality (ϑ), with units angular degrees. As morphologies of individual mitochondria are heterogeneous within a cell and among cell types [3], we introduced a continuous range of mitochondrial masses, from a minimum value (M_min_) of 0.5 μm^2^ [31] to a maximum value (M_max_) of 3 μm^2^ [32, 33] (Fig 1A).

#### Mitochondrial movement rule set

In order to prevent unfeasible actions such as overlaps, each mitochondrion first checks its surrounding area within a radius of 1.0 μm. If this area is free, the mitochondrion first moves with a velocity v following its current directionality ϑ. In each time iteration, the velocity v is randomly chosen within pre-set intervals depending on the intracellular position as explained below. After movement, the directionality is updated by choosing random angle ϑ_1_ between 0° and 360° (Fig 1C). If the surrounding area is occupied by another mitochondrion (without fusing with it), the nucleus, or the cell border, the mitochondrion does not move and its directionality is updated by choosing a random angle ϑ_2_ between 0° and 90° (Fig 1C).

Mitochondrial mobility can vary in relation to its position inside the cell [34], as the average velocity of mitochondria in the cytosolic region is faster (0.22–0.50 μms^-1^) than of mitochondria localized in the perinuclear region (0.10 μms^-1^) [33, 35–38]. Therefore, to simulate mitochondrial spatial dynamics, we subdivided the cell into two regions (Fig 1A). The first region corresponds to the perinuclear area (diameter ≤ 25 μm), where mitochondria were assigned low velocity magnitudes (v_l_) in the interval 0 ≤ v_l_ ≤ 0.44 μms^-1^. The second region corresponds to the bulk cytosolic area, where mitochondria were assigned high velocity magnitudes (v_h_) in the interval 0 ≤ v_h_ ≤ 1 μms^-1^. Finally, since small mitochondria move faster than large mitochondria [1, 39], we assumed that mitochondrial velocities are inversely proportional to the mass, i.e., v = v*/m, where v* assumes a value of v_l_ or v_h_ according to the intracellular position.

#### Mitochondrial fusion rule set

Mitochondrial fusion kinetics was parameterized in terms of a fusion probability (F_p_), with higher F_p_ values relating to larger fusion rates. A given mitochondrion can fuse with another mitochondrion according to the following two rules. (I) Before moving, a random number is sampled between 0 and 100. If this number is below the value of F_p_, the mitochondrion is allowed to fuse given that there is another mitochondrion within an area of twice its own radius. (II) To avoid formation of unrealistically large mitochondria, the fusion process only takes place if the sum of the mass of the two fused mitochondria is below a maximal mitochondrial mass (M_max_) (Fig 1D and S1 Fig).

#### Mitochondrial fission rule set

Mitochondrial fission kinetics were parameterized in terms of a fission probability (f_p_). A given mitochondrion can undergo fission according to the following two rules. (I) A random number is sampled between 0 and 100, and if this number is below the value of f_p_, the mitochondrion splits into two daughter mitochondria. (II) To avoid unrealistically small mitochondria, the fission process can only occur in mitochondria of a mass greater than two times the minimum mass (M > 2M_min_) (Fig 1D and S1 Fig). The mass of the parent mitochondrion is distributed randomly among the two daughters, satisfying mass conservation and bounds as follows:

$$M_{1}=\left(X_{F}\cdot \left(\frac{1}{2}-\frac{M_{min}}{M}\right)+\frac{M_{min}}{M}\right)\cdot M$$ (1)

$$M_{2}=M-M_{1}$$ (2)

Where M is the mass of the parent mitochondrion, M_1_ and M_2_ are the masses of the smallest and biggest daughter mitochondrion, respectively, and X_F_ is a scaling coefficient estimated uniformly in the interval [0,1] (X_F_ ~ U[0,1]) (S1 Fig).

Notably, mitochondrial fusion and fission cycles occur on the order of minutes [40–42], and thus, at larger time scales than mitochondrial movement. To capture these time scale differences, the rule sets for mitochondrial fission and fusion were only evaluated every 5 minutes [43, 44], while the rule set for mitochondrial movement was evaluated at all time iterations. The update frequency of fusion and fission rules is denoted here as fusion frequency F_f_ and fission frequency f_f_, respectively, both of them set to 1 event per 5 minutes for all simulations [45, 46] (Fig 1B and 1D, S1 Fig and S1 Code). Table 1 summarizes all model parameters and their respective value.

**Table 1:** Description of the parameters used in the model.

Parameter	Description	Value	Biological evidence
D_C_	Cell diameter.	50 μm	2–100 μm human cell [31].
D_N_	Nucleus diameter.	16.6 μm	2–10 μm human cell [31].
M_min_	Minimum mitochondrial mass.	0.5 μm^2^	Mitochondrial morphologies vary within the cell [3, 47]. Length: 1–10 μm mammalian cell [31]. Area: 2–10 μm^2^ myelinated axon [33], 0.1–0.2 μm^2^ HeLa cell [48].
M_max_	Maximum mitochondrial mass.	3 μm^2^	
v_l_	Mitochondrial velocity in the perinuclear region.	[0,0.44] μms^-1^	0.5 μms^-1^ along cytoskeleton, 0.22 μms^-1^ perinuclear region, BY-2 cell [35]. 0.24–0.69 μms^-1^ axon [37]. 0.6 μms^-1^ myelinated axon [33].
v_h_	Mitochondrial velocity in the cytosolic region.	[0,1] μms^-1^	
ϑ_1_	Directionality. Rotational angle of mitochondria in free space.	[0°,360°]	Changes in the directionality vector allow full range of directional movements.
ϑ_2_	Directionality. Rotational angle of mitochondria constrained by other agents.	[0°,90°]	Restricts movements in crowded conditions to avoid unfeasible actions.
Initial mitochondrial mass	Total mitochondrial mass at time point 0.	300 μm^2^	Mitochondrial volume percentage of HeLa cells: 10%-15% [48, 49] and 20.1%-44.6% [49].
Fusion probability (F_p_)	Probability of fusion.	[0,100] %	Fusion events can vary according to the cellular state [47]. In our model this probability varies (is enhanced) according to the level of cellular stress.
Fission probability (f_p_)	Probability of fission.	[0,100] %	Fission events can vary according to the cellular state [47]. In our model this probability varies (is reduced) according to the level of cellular stress.
Fusion frequency (F_f_)	Frequency of fusion.	5 min	Frequency of fusion/fission events per mitochondrion in COS7 and INS1 cells: ~1 event per 5–20 min [43, 46].
Fission frequency (f_f_)	Frequency of fission.	5 min	Frequency of fusion/fission events per mitochondrion in COS7 and INS1 cells: ~ 1 event per 5–20 min [43, 46].
Biogenesis probability (B_p_)	Probability of biogenesis.	[0,100] %	Biogenesis is upregulated by bioenergetic stress and nutrient deprivation [4]. In our model this probability varies (is enhanced) according to the level of cellular stress.
Biogenesis frequency (B_f_)	Frequency of biogenesis.	28.9 min	Biogenesis occurs according to cellular needs [4]. Value estimated by maximizing Eq 4, optimizing the repopulation of mitochondria in a cell, starting from mitochondrial mass equal 1 to 15% of the cell area (mass) within 2 days.
Damage probability (D_p_)	Probability of damage.	[0,100] %	Damaging events can vary according to different external/internal cell factors [5–7, 47].
Degradation frequency (D_f_)	Frequency of degradation of damaged mitochondria.	5.7 min	Quality control mechanism to eliminate damaged mitochondria [5–7, 47]. Value estimated by maximizing Eq 5, optimizing the complete depletion of damaged mitochondria within one day starting from a damaged mitochondrial mass of 15% of the cell area.
Ratio *low/high* damage	Probability of having a low or high damage.	[0,100] %	Mitochondrial damage levels vary according to mitochondrial activity [47].
Damage threshold (d_T_)	Threshold for a low damaged mitochondrion to become highly damaged.	12.4 min	Value estimated by maximizing Eq 5, optimizing the complete depletion of damaged mitochondria within one day starting from a damaged mitochondrial mass of 15% of the cell area.
MR threshold (MR_T_)	Mitophagy receptor threshold for autophagosome formation.	12 min	Autophagosome formation for clearance of damaged mitochondria [5–7, 47]. Value estimated by maximizing Eq 5, optimizing the complete depletion of damaged mitochondria within one day starting from a damaged mitochondrial mass of 15% of the cell area.
Δt	Time step increment.	1 sec	Increment of the simulations time steps.
Number of cells	Number of cells considered in each simulation.	100	Number of cells considered to account for cell-to-cell variability.

### The effect of fusion and fission on mitochondrial morphology is intrinsically connected to the total mitochondrial mass

**Fig 2:**
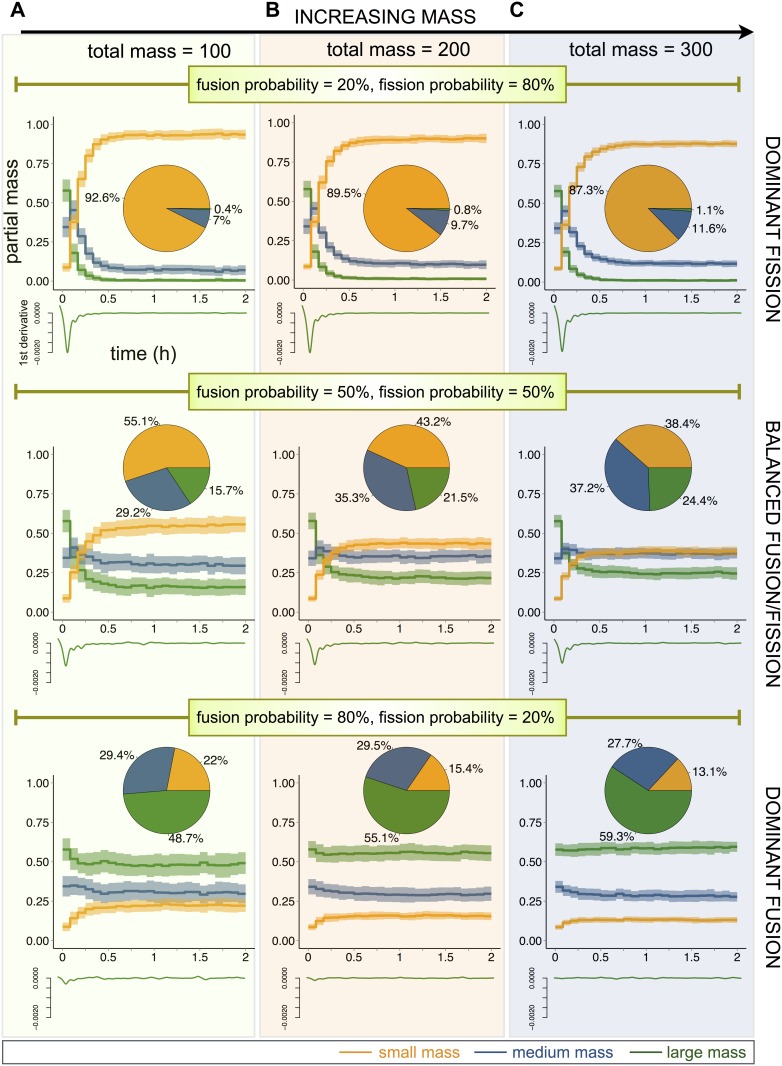
Impact of fusion and fission probabilities and mitochondrial mass on mitochondrial subpopulations. (A) Time course response of mitochondrial subpopulations for low total mass (*M* = 100; 5% of the whole cell area). Three mitochondrial subpopulations are considered: small mass (orange line), medium mass (blue line) and large mass (green line). The partial mass reports the sum of the masses from all mitochondria in a given subpopulation divided by the total mitochondrial mass. Lines display the mean among 100 simulations, and shaded regions display the standard deviation. Small graphs underneath show the evolution of the first derivative of mitochondria with large mass (green line). Three different fusion/fission probability rates are used: 20%/80%, 50%/50% and 80%/20%. Pie charts report the partial mass (in terms of percentages) for the subpopulations at the final simulation time (2 hours). (B) Same as (A) but with a total mitochondrial mass of 200 (10% of the whole cell area). (C) Same as (A) but with a total mitochondrial mass of 300 (15% of the whole cell area).

We first studied how the combined effect of variations in mass and fusion/fission kinetics affect the mitochondrial population. To this end, simulations were performed using three combinations of fusion-fission probabilities, i.e. dominant fission (F_p_ = 20%, f_p_ = 80%), dominant fusion (F_p_ = 80%, f_p_ = 20%), and balanced fusion-fission (F_p_ = f_p_ = 50%). As mitochondrial masses range from approximately 10–40% of cellular volume [48] we simulated three total mitochondrial masses: 100 μm^2^ (5% of the total cell area), 200 μm^2^ (10% of the total cell area) and 300 μm^2^ (15% of the total cell area) (Fig 2A–2C and S1 Movie). Simulations were run for 2 hours and were initialized by randomly distributing the total mitochondrial mass among a population of mitochondria with masses between M_min_ and M_max_.

To analyze simulations, we classified mitochondria into three subpopulations: small (M ≤ 1 μm^2^, color orange), medium (1 μm^2^ < M < 2 μm^2^, color blue) and large (M ≥ 2 μm^2^, color green) (Fig 1A). To evaluate the distribution of mitochondria among subpopulations, a partial mass was estimated for each subpopulation by adding the masses of all mitochondria in the subpopulation divided by the total mass. For each set of parameters, the average of 100 simulations is reported, where each simulation is interpreted as one stochastic cell. To indicate the transition to steady state, the first time-derivative was calculated for the large mass subpopulation (green trace). Pie charts report average subpopulations distributions at the final time point (2 hours).

As indicated by the first time-derivatives, mitochondrial subpopulation distributions stabilized within approximately 15 minutes (Fig 2A–2C). For all masses, under conditions of dominant fission, mitochondrial populations adopted a fragmented state (87% to 92% small subpopulation). In contrast, under conditions of dominant fusion, the large subpopulation was most abundant (49% to 69%). Notably, increasing the total mitochondrial mass further increased the abundance of large mitochondria.

These results suggest that mitochondrial morphology homeostasis depends on both, fusion and fission probabilities and the total mitochondrial mass. With low total masses (M = 100 μm^2^), the fusion process is limited by the probability that two mitochondria are in proximity with each other. We suggest then that for low masses, the cell has very little control over the morphology of its mitochondrial network. Alternatively, at masses equivalent to 15% of the cell area (M = 300 μm^2^), which corresponds to the mitochondrial content of a typical cell line (e.g. HeLa) [49], physical proximity is less limiting. Therefore, higher mitochondrial mass grants the cell better controllability over its mitochondrial network morphology.

Importantly, variations in the total mass of mitochondria affect both heterogeneity of the intracellular mitochondrial population and cell-to-cell variability. Namely, higher masses and balanced probabilities of fusion and fission allow for more heterogeneous populations with mitochondria of different sizes, as indicated by the distributions on the pie charts (Fig 2C, middle graph). On the other hand, higher masses lead to less cell-to-cell variability with respect to smaller ones, as can be observed from the spread of the partial mass in the time course plots (Fig 2A–2C).

### Influence of mitochondrial directionality on subpopulation distributions

**Fig 3:**
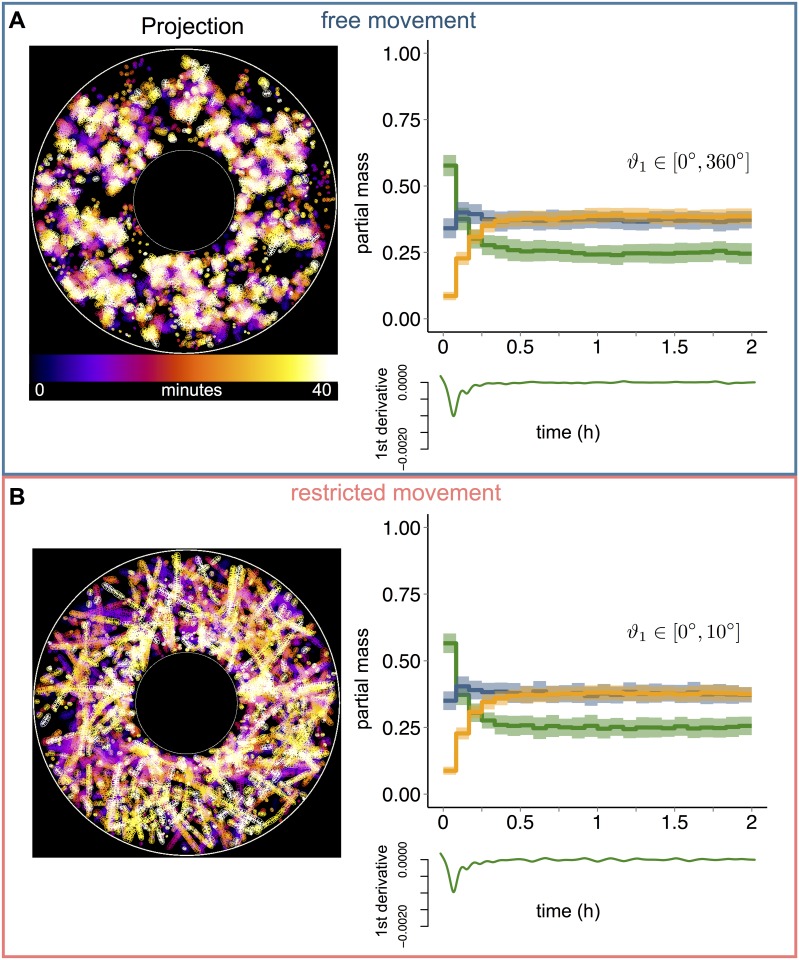
Impact of mitochondrial directionality on mitochondrial mass subpopulation distributions. (A) Mitochondrial movements are indicated by projecting 25 images, one every 100 time steps, representing a total of ~40 minutes of a simulation. Time points are indicated according to color-code. Line graphs display mean values and standard deviation (shaded regions) of 100 simulations for mitochondrial population with free movement. The initial mass value was fixed to 300 and the angle ϑ_1_ ϵ [0°, 360°]. The plot represents the evolution of three mitochondrial subpopulations, small mass (orange line), medium mass (blue line) and large mass (green line), normalized to the total mass at time point 0. Small graphs underneath show the evolution of the first derivative of mitochondria with large mass (green line). The simulations were performed for a total time of 2 hours. (B) Same as (A) but with restricted movement, ϑ_1_ ϵ [0°, 10°].

Mitochondrial movement is essential to meet energy demands and prevent cell death [50], and is coordinated by different components of the cytoskeleton such as microtubules [51], which mediate directed movement of organelles [52]. Therefore, we sought to investigate the effect of directed vs. free movement on the behavior of the mitochondrial subpopulations. To this end, we modified the range of variation of the directionality property of mitochondrial agents, assigning unrestricted rotation values (ϑ_1_ ϵ [0°, 360°]) (Fig 3A), and restricted rotation values (ϑ_1_ ϵ [0°, 10°]) (Fig 3B and S2 Movie). The total mass was set to 300, and fusion and fission probabilities to 50%.

Compared to unrestricted movement (Fig 3A), restricted directionalities (Fig 3B) resulted in mitochondrial trajectories that followed straight lines, effectively mimicking directed movements. However, the time course distribution of mitochondrial subpopulations was unchanged between restricted and unrestricted movements. These findings suggest that the impact of mitochondrial directionality on the state of the mitochondrial population is negligible.

### Transmission dynamics of the mitochondrial network

**Fig 4:**
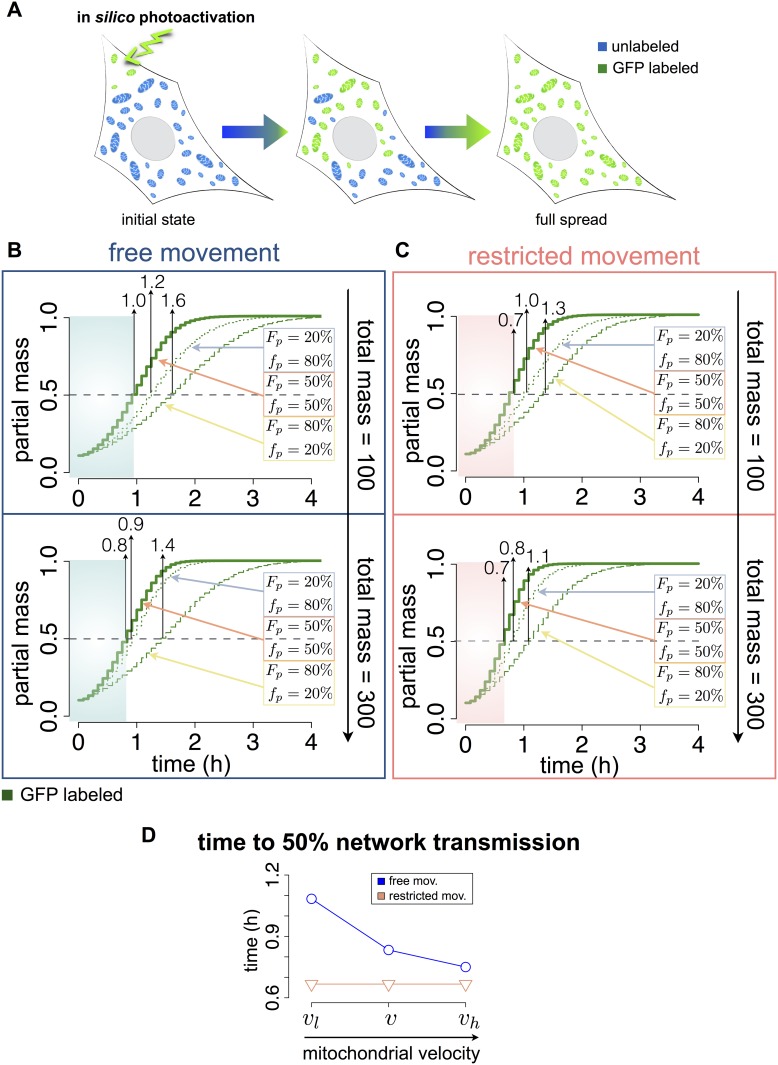
In silico photoactivation to quantify transmission dynamics in mitochondrial networks. (A) Schematic representation of an *in silico* photoactivation. 10% of mitochondrial mass is assigned a GFP label, which transmit through the cell upon a fusion event: fusion of a mitochondrion with a GFP-labeled mitochondrion, results in a parent GFP-labeled mitochondrion, which upon fission results in two GFP-labeled daughter mitochondria. (B) Transmission dynamic of photoactivated GFP across the mitochondria population moving with free motion (ϑ_1_ ϵ [0°, 360°]), starting from 10% of labeled mitochondria (green). Simulations were performed with total mitochondrial masses of 100 and 300 and three different fusion/fission probability rates: F_p_/f_p_ = 20%/80% (dotted line), F_p_/f_p_ = 50%/50% (solid line) and F_p_/f_p_ = 80%/20% (dashed line). 100 simulations were performed each and plots represent the average values normalized to the total mass at time point 0. Blue regions indicate the time to 50% network transmission with fusion/fission probability rates of 50%/50%. (C) Same as (B) but with restricted movement, ϑ_1_ ϵ [0°, 10°]. Pink regions indicate the time to 50% network transmission with fusion/fission probability rates of 50%/50%. (D) Effect of the mitochondria mobility on transmission dynamics in the case of free movement (blue circles, line) and restricted movement (pink triangles, line). Detailed representation of the time needed to the GFP labeled mitochondrial population to exceed the unlabeled population for an initial total mitochondrial mass of 300 with a fixed fusion/fission probability rate of 50%/50% and three different velocities: v_l_ ϵ [0, 0.44] μms^-1^, v_h_ ϵ [0, 1] μms^-1^ and v, equal to v_l_ in the perinuclear region and to v_h_ outside.

We next investigated how fusion and fission dynamics, directionality and mobility impact the mitochondrial network transmissivity [41], defined here as the ability to transmit signals among the mitochondrial population by consecutive fusion-fission events. Mitochondrial network transmissivity has been previously measured by photoactivatable GFP within a subpopulation of mitochondria (~10%), finding that the complete mitochondrial population was fluorescent within 30–60 minutes [53, 54]. Here, to simulate transmission dynamics, we introduced the following rule set for inter-mitochondrial GFP heritability: (I) fusion of an unlabeled mitochondrion with a GFP-labeled mitochondrion, results in a parent GFP-labeled mitochondrion. (II) Fission of a GFP-labeled mitochondria results in two GFP-labeled daughter mitochondria. We simulated the effect of a localized photoactivation of 10% of the cellular mitochondria, and quantified GFP transmission through the cell for several values of mitochondrial mass and fusion/fission probabilities (Fig 4A).

Simulations were performed with total mitochondrial masses of 100 and 300, fusion/fission probability ratios of 20%/80%, 50%/50% and 80%/20%, and unrestricted (ϑ_1_ ϵ [0°, 360°]) (Fig 4B and S3 Movie) or restricted movement (ϑ_1_ ϵ [0°, 10°]) (Fig 4C). To quantify network transmissivity, we calculated the time to transmit the GFP signal to 50% of mitochondrial mass, denoted as 50% transmission time (shaded areas in Fig 4B and 4C).

Under conditions of free movement (Fig 4B), a balanced probability of fusion and fission (50%/50%) produced the fastest GFP transmission (smallest 50% transmission time), while a fusion/fission probability of 80%/20% showed the slowest transmission. Moreover, by increasing total mass, the 50% transmission time was slightly reduced (Fig 4B, blue regions). Similar tendencies were obtained under conditions of restricted movement, but the 50% transmission time was decreased relative to free movement (Fig 4C, **pink regions**).

We further examined the impact of mitochondrial mobility on network transmissivity by changing the velocity intervals of mitochondria (Fig 4D). Mitochondria were assumed to (I) adopt low velocities in all the cellular spatial domain (v_l_ ∀ x), (II) adopt high velocities in all spatial domain (v_h_ ∀ x) or (III) adopt low velocities in the perinuclear region and high velocities in the bulk cytosol (v = v_l_, or v = v_h_),as it was implemented in the previous sections. Simulations were performed using fusion and fission probabilities of 50%, a total mass of 300 μm^2^, and either free or restricted mitochondrial directionalities, and the 50% transmission time was calculated (Fig 4D). Notably, under conditions of restricted movement (pink line), decreasing or increasing velocities had no impact, and compared to free movement, GFP transmission was considerably faster. In contrast, under conditions of free movement, increasing velocities reduced the 50% transmission time (blue line). These findings suggest that restricted mitochondrial movements suppress sensitivities to alterations in spatial dynamics. Therefore, all subsequent simulations were performed under conditions of free mitochondrial movements. Overall, simulation results indicate that fusion and fission events and levels of mitochondrial mass together determine morphology distributions, while the transmission of signals across the mitochondrial population is determined by fusion and fission events and mitochondrial velocities.

Given these distributed contributions, we subsequently sought to quantify the impact of parameter perturbations. Sensitivity analysis is an indispensable procedure to test and tune *in silico* experiments, since it emphasizes the role and the biological relevance of the parameters involved [55]. As an essential tool able to evaluate and refine mathematical models [56], we applied a global sensitivity analysis using Latin-Hypercube Sampling (LHS) and Extended Fourier Amplitude Sampling Test (eFAST) [57].

**Fig 5:**
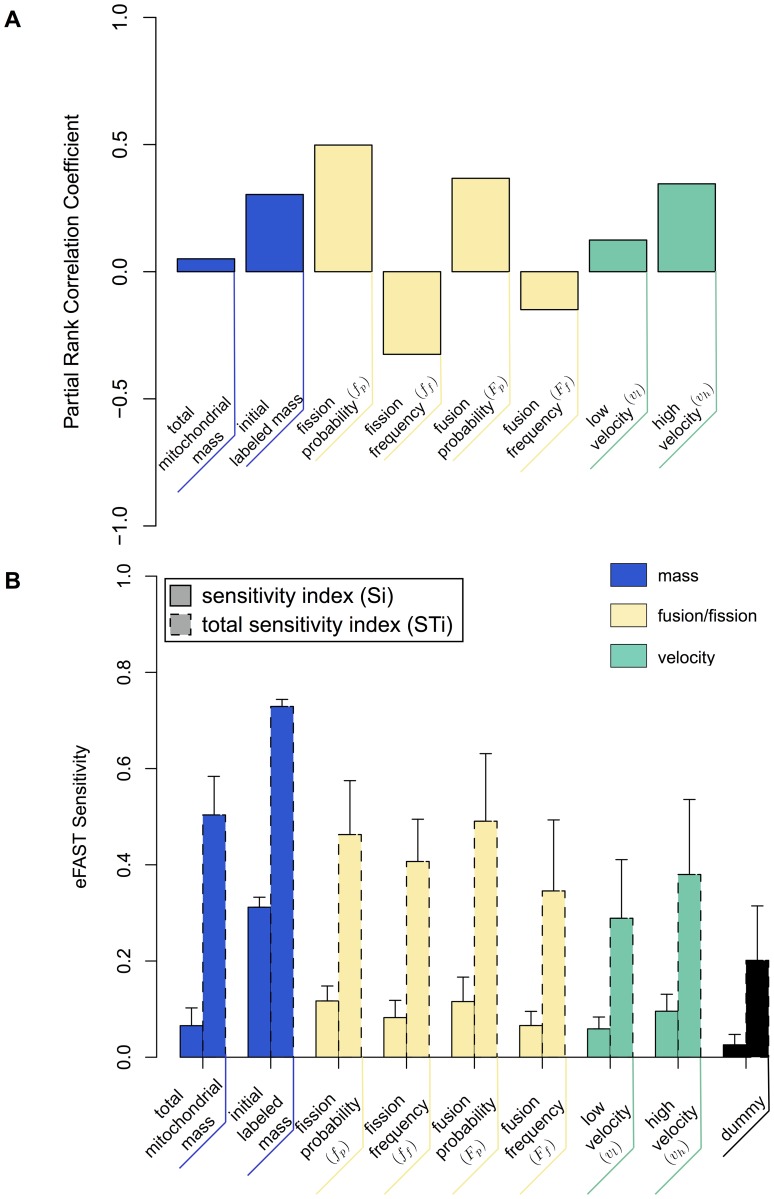
Sensitivity analysis of the transmission dynamics model. (A) Effect of parameters perturbation on transmission dynamic. Bar graph represents Partial Rank Correlation Coefficient values for each parameter-output response pairing resulting from a Latin-hypercube sampling of 500 parameter value sets (parameter ranges are described in section “Transmission dynamics of mitochondrial network connectivity”). For each parameter set the model was performed over 6 hours. (B) Results of the eFast method used to partition variance in simulation results between parameters. Solid Bars: sensitivity index (Si)–the fraction of output variance explained by the value assigned to that parameter when parameter of interest; Dashed Bars: total sensitivity index (STi)–the variance caused by higher-order non-linear effects between that parameter and the other explored (includes value of Si). Error bars are standard error over four resample curves. Parameter values sets were generated using the sinusoidal sampling approach. 65 parameters were obtained from each of the 4 resampling curves, producing 2080 parameter value sets (260 per parameter).

We concentrated on seven parameters, each constrained to the following respective ranges: fusion frequency (0–30), fusion probability (0–100), fission frequency (0–30), fission probability (0–100), mitochondrial mass (50–500 μm^2^) and mitochondrial velocities in bulk cytosol v_h_ (0.01–1 μms^-1^) and in the perinuclear region v_l_ (0.01–1 μms^-1^). Specifically, we sought to assess how parameter perturbations impact the mass of GFP-labeled mitochondria. Using the Latin-Hypercube sampling approach, parameters were simultaneously perturbed, to identify parameters that are highly influential on simulation behavior. Five hundred parameter value sets were generated from the parameter space and simulated (each for the duration of 6 hours). For each parameter, median response values (evolution over time of the GFP-labeled mitochondrial population) were plotted against corresponding parameter value used (S2 Fig), and partial rank correlation coefficients were calculated (Fig 5A).

The correlation coefficients (Fig 5A and S2 Fig) indicate that the total mitochondrial mass and low-perinuclear velocity (v_l_) have the least impact on the network transmission, and that fusion and fission-related parameters and high-cytoplasmic velocity (v_h_) have distributed control over transmission. Positive correlation coefficients indicate that increases in fission probability, fusion probability and velocity contribute to a faster transmission dynamics. In contrast, negative correlations for fusion and fission frequencies indicate that their decrease contributes to an increased transmission. Additionally, the positive correlation between fusion and fission probabilities with the population of GFP-labeled mitochondria, emerged mostly for lower ranges and stabilized for values greater than ~60% (S2C and S2E Fig).

We further utilized eFAST global sensitivity analysis to identify highly influential parameters. For nine parameters (eight plus the ‘dummy’ used as negative control), we generated parameter value sets using the sinusoidal curve sampling approach. Briefly, 65 parameter values from each curve were produced, and employed four resampling curves, producing 2080 parameter value sets, 260 per parameter. Simulation responses were analyzed using the Fourier frequency approach and median responses (evolution over time of the GFP-labeled mitochondrial population) calculated [57]. Plots were created detailing the first-order (Si) and total-order (STi) sensitivity indexes calculated for each parameter (Fig 5B). To quantify the impact of a parameter on the simulation, we used the “initial-labeled mass” as a positive control, and as a negative control “dummy”, a parameter which has an arbitrary value range but no actual impact on model behavior. Note, “dummy” parameters result in non-zero sensitivity indexes due to noise derived from the intrinsic stochasticity of the model [57, 58]. Compared to “initial-labeled mass”, eFAST analysis indicates an overall equilibrium of 7 out of 8 parameters, since variations in the Si and STi sensitivity indexes between parameters is low. This indicates the current state of the model is not over-parametrized.

### Integrating mitochondrial dynamics with mitochondrial biogenesis

So far, our model captured essential spatial and temporal behavior of mitochondrial morphology and mobility dynamics at a time scale of seconds-to-hours assuming a constant total mitochondrial mass. However, mitochondrial populations are not a closed systems, and on a time scale of hours to days mitochondrial biogenesis occurs from growth and division of pre-existing mitochondria [34]. Furthermore, over time and in response to stress, mitochondria become dysfunctional and quality control of mitochondria occurs through the process of mitophagy [47]. We therefore subsequently incorporated biogenesis and mitophagy behavior.

**Fig 6:**
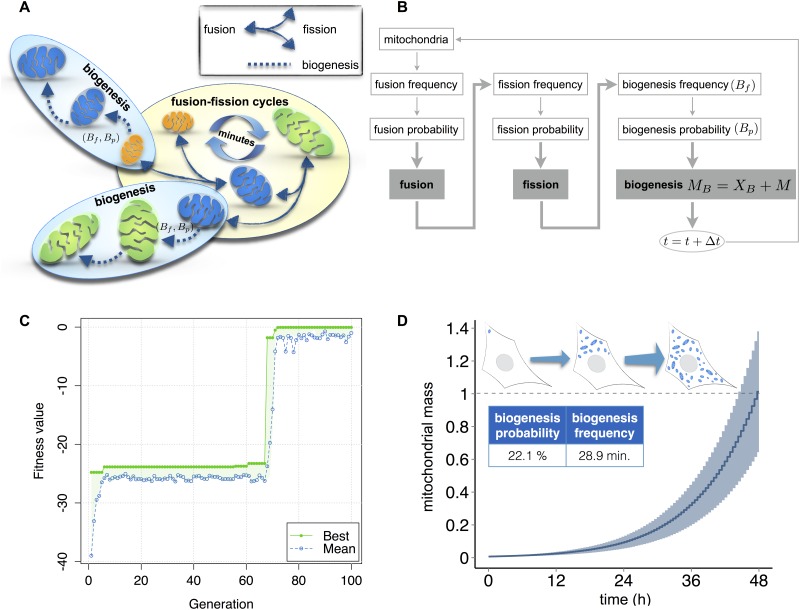
Integration of mitochondrial biogenesis through parameter fitting. (A) Schematic representation of mitochondrial fusion, fission and biogenesis events. Biogenesis is represented by frequency (B_f_) and probability (B_p_) parameters, and can occur in all mitochondria with a mass less than M_max_ = 3.0 μm^2^. (B) Basic flow chart of the model representing fusion, fission and biogenesis events. Shaded boxes indicate the main processes of the model (fusion, fission and biogenesis) with governing equation of the biogenesis process. Time step used in all the simulation, Δt = 1 sec. (C) Maximization of the fitness function O_B_ within 100 iterations, reaching a value of -0.049, using a classic genetic algorithm (settings described in the Methods section). (D) Mean of 100 simulations of mitochondrial mass growth over 48 hours, beginning with a mitochondrial mass of 1 and using the parameter values obtained from the optimization (C), biogenesis probability = 22.1% and biogenesis frequency = 28.9 minutes.

We first established a rule set to describe mitochondrial biogenesis (Fig 6A). Sub-maximal mitochondria (M < 3 μm^2^) undergo a growth event based on a biogenesis probability (B_p_), which can occur according to a biogenesis frequency (B_f_) (Fig 6B). All mitochondria grow by increasing their actual mass M to a new value M_B_, according to the following relation:

$$M_{B}=X_{B}+M$$ (3)

Where X_B_ ~ U[0, M·50%]. In this way, every mitochondrion can at most double its mass during one growth event. Additionally, in order to avoid unrestricted growth, the resulting mass of the biogenesis process M_B_ can not exceed a preset maximum value M_max_.

Different lines of evidence give insight into mitochondrial biogenesis rates. Biogenesis can double the population of mitochondria during a typical cell division cycle of 48 hours [59], and following near depletion, mitochondrial repopulation occurs over days [60, 61]. Moreover, activated mitochondrial biogenesis increases mitochondrial markers by approximately 2–4 fold [62]. From these biological constraints, using a genetic algorithm [63], we optimized biogenesis frequency and biogenesis probability parameters. Starting with a total initial mass of one, fusion and fission probabilities of 50%, and fusion and fission frequencies of 5 minutes, we optimized biogenesis frequency and biogenesis probability, in order to reach a total mitochondrial mass equal to 300 μm^2^ within 48 hours. Therefore, we maximized the following objective function (O_B_):

$$O_{B}=-\left|T_{F}-48\right|$$ (4)

Where T_F_ represents the final simulation time, i.e., the time needed to reach a total mass of 300 μm^2^.

The genetic algorithm was able to achieve a final fitness function value of -0.049 within 100 iterations (Fig 6C), generating a biogenesis frequency of 28.9 minutes and a biogenesis probability of 22.1%. With these optimized values, the mass of mitochondria increases from 1 to 15% of the cell within two days (Fig 6D and S4 Movie). Note, with these parameters doubling of the mitochondrial population following cell division occurs in approximately 9 hours (S3 Fig), which is less than, and therefore compatible with, the HeLa cell division cycle [64].

### Integrating mitochondrial dynamics with mitochondrial damage and mitophagy

**Fig 7:**
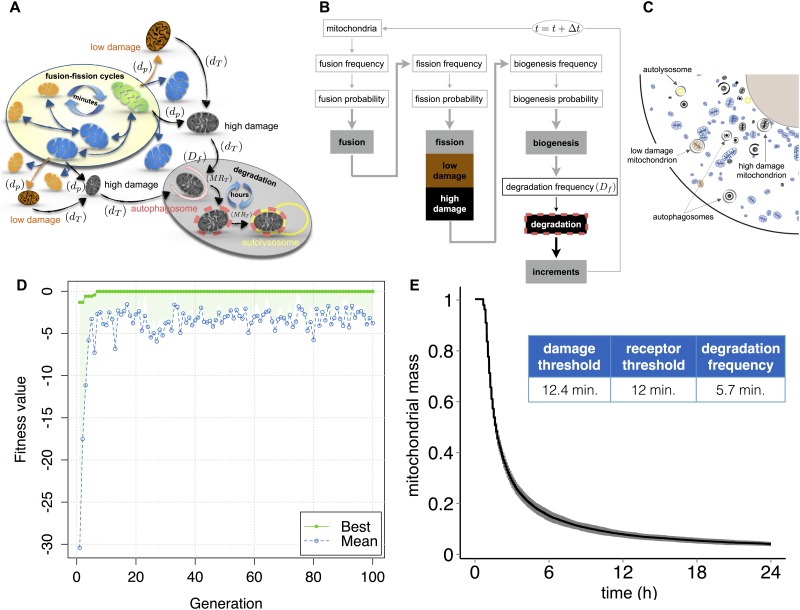
Integration of mitochondrial damage sensing and mitophagy through parameter fitting. (A) Schematic representation of mitochondrial fusion and fission cycles, damage and mitochondrial degradation events. Degradation is represented by frequency (D_f_) parameter and damage (d_T_) and receptor (MR_T_) thresholds. (B) Basic flow chart of the model representing fusion, fission, biogenesis and degradation events. Shaded boxes indicate the main processes of the model (fusion, fission, biogenesis, *low/high* damage, degradation and increments). Time step used in all the simulation, Δt = 1 sec. (C) Spatial model representation. Highly-damaged mitochondria are represented in black and mitochondria with low damage in brown. The yellow circle indicates an autolysosome and the black circle and semicircle indicate stages of autophagosome formation. (D) Maximization of the fitness function O_D_ within 100 iterations, reaching a value of -0.02, using a classic genetic algorithm. Algorithm (settings described in the Methods section). (E) Mean of 100 simulations of degradation of damaged mitochondria over 24 hours, beginning with 50% *low* damaged and 50% *high* damaged mitochondria and using the parameters obtained from the optimization (D), receptor threshold = 12 minutes, damage threshold = 12.4 minutes and degradation frequency = 5.7 minutes. The initial total mass consists of two equal parts of high-damaged and low-damaged mitochondria.

We next implemented quality control of damaged mitochondria by mitophagy [6, 8, 65, 66], using the parameter-fitting approach developed for mitochondrial biogenesis (Fig 7A) and a rule-set describing mitochondrial damage and removal (Fig 7B and S4 Fig). As mitochondria utilize fission to segregate healthy from damaged mitochondria [47], we included a probability variable (d_p_) for the fission process whereby one mitochondrion fragments into one healthy and one damaged mitochondrion [43]. Furthermore, we specified two damage states: *low* and *high*, corresponding to pre-mitophagy and mitophagy induction states [8]. To simulate the mitochondrial capture and degradation, we introduced two autophagy agents: sequestering autophagosomes and degrading lysosomes (Fig 7C), which undergo a multi-step interaction with mitochondria to approximate mitophagy dynamics. Over time, *low* damage levels increase, until levels exceed a fixed-damage threshold (d_T_), and become *high* damaged. Highly-damaged mitochondria acquire a mitophagy receptor (MR) level, which is updated at every time step. To approximate the sequestration process, once a receptor threshold is reached, an autophagosome forms at the damaged mitochondrion (Fig 7A) and the receptor level is again set to zero. Subsequently, when the receptor level exceeds threshold a second time; the mitochondrion is considered irreversibly sequestered. Concurrently, a mobile, degradative lysosome is randomly generated within the cell, and the lysosome is attracted to sequestered mitochondrion and both are degraded upon contact. All the above rules are evaluated according to a degradation frequency (D_f_). The parameters receptor level and damage level are updated and increased at every time step.

Upon full mitophagy activation, mitochondrial populations can be eliminated by mitophagy within 1–2 days [67, 68]. Based on these measurements we optimized three parameters, receptor threshold, damage threshold and degradation frequency, to deplete the population of mitochondria (total mass of 300 μm^2^). Simulations were initialized by setting 50% of the mitochondrial mass to a *low* damage state and 50% to a *high* damage state. The following objective function (O_D_) was maximized:

$$O_{D}=-\left|T_{F}-24\right|$$ (5)

Where T_F_ represents the final simulation time, i.e., the time needed to achieve degradation of the total mitochondrial population.

The genetic algorithm achieved a final fitness function value of -0.02 within 100 iterations (Fig 7D) generating a receptor threshold of 12 minutes, a damage threshold of 12.4 minutes and a degradation frequency of 5.7 minutes. The resulting model fit eliminates all damaged mitochondria within one day (Fig 7E and S5 Movie).

### Energetic sensing, mitophagy and mitochondrial biogenesis must be considered as integrated processes

**Fig 8:**
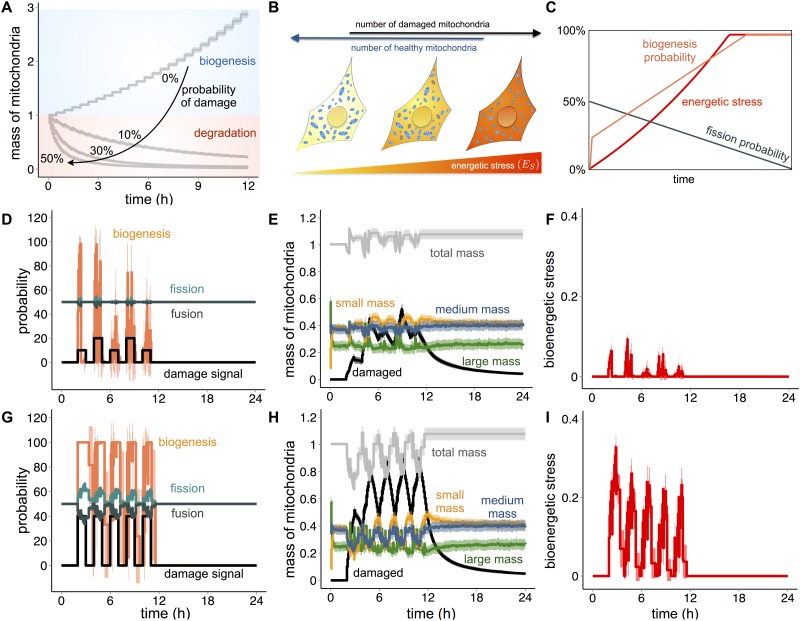
Interaction between mitochondrial health and cellular energetic state, based on the energetic stress. (A) Evolution of mitochondrial mass (grey lines) over time subjected to four different damage signals (black line). Line graphs display mean value and standard deviation (shaded regions) of 100 simulations for an initial total mitochondrial mass of 300 normalized to the total mass at time point 0. Parameter values obtained from the optimization were used, in particular, fusion probability = fission probability = 50%, fusion frequency = fission frequency = 5 minutes, biogenesis probability = 22.1%, biogenesis frequency = 28.9 minutes, receptor threshold = 12 minutes, damage threshold = 12.4 minutes, degradation frequency = 5.7 minutes and damage probabilities: 0%, 10%, 30% and 50%. Simulations were performed for a total of 12 hours. Blue region indicates the area in which biogenesis is dominant while red region indicates the area in which degradation is dominant. (B) Schematic representation of the link between the number of healthy and damaged mitochondria with the energetic stress (E_S_) of the cell. (C) Schematic representation of the connection between energetic (red line) stress and fission (dark green line) and biogenesis (orange line) probabilities. (D) Line graphs display mean value and standard deviation (shaded regions) of 100 simulations of probabilities of fusion (dark green line), fission (light green line), biogenesis (orange line) and damage signal (black line). Initial values assigned to the model: fusion probability = fission probability = 50%, fusion frequency = fission frequency = 5 minutes, biogenesis probability = 22.1%, biogenesis frequency = 28.9 minutes, receptor threshold = 12 minutes, damage threshold = 12.4 minutes, degradation frequency = 5.7 minutes. All the simulations were performed for a total time of 24 hours. (E) Line graphs display mean value and standard deviation (shaded area) of 100 simulations for an initial mitochondrial mass of 300 of the total mitochondrial mass (grey line) and three mitochondria subpopulations: small mass (orange line), medium mass (blue line) and big mass (green line) subjected to five different damage signals (10% and 20%) every two hours for one hour. The plot represents the evolution of the total mass and of three mitochondrial subpopulations normalized to the total mass at time point 0. Initial values assigned to the model: fusion probability = fission probability = 50%, fusion frequency = fission frequency = 5 minutes, biogenesis probability = 22.1%, biogenesis frequency = 28.9 minutes, receptor threshold = 12 minutes, damage threshold = 12.4 minutes, degradation frequency = 5.7 minutes. All the simulations were performed for a total time of 24 hours. (F) Line graphs display mean value and standard deviation (shaded regions) of 100 simulations of bioenergetics stress (red line). Initial values assigned to the model: fusion probability = fission probability = 50%, fusion frequency = fission frequency = 5 minutes, biogenesis probability = 22.1%, biogenesis frequency = 28.9 minutes, receptor threshold = 12 minutes, damage threshold = 12.4 minutes, degradation frequency = 5.7 minutes. All the simulations were performed for a total time of 24 hours. (G) Same as (D) but with mitochondria subjected to five different damage signals (40%) every two hours for one hour. (H) Same as (E) but with mitochondria subjected to five different damage signals (40%) every two hours for one hour. (I) Same as (F) but with mitochondria subjected to five different damage signals (40%) every two hours for one hour.

With the parameters obtained from the optimization of biogenesis and mitophagy, our model can simulate the mitochondrial repopulation within two days, and remove a population of damaged mitochondria within one day, respectively. However, when these processes are combined, in the absence of damage, biogenesis is dominant and uncontrolled growth occurs (Fig 8A). In contrast, if we introduce a fixed probability of damage, the total mass of healthy mitochondria eventually decays over time, in a manner proportional to the probability of damage, and the model fails to maintain mitochondrial homeostasis. This split between biogenesis and degradation dominant regions reveals instability in the system, suggesting biogenesis and degradation must be considered as integrated processes. We thus considered functions of the metabolic sensor AMP-activated protein kinase (AMPK). AMPK is activated in response to decreased ATP production, i.e. bioenergetic stress [69], and activates PGC-1alpha [70], the master regulator of mitochondrial biogenesis [71]. Moreover, AMPK regulates the removal of defective mitochondria [72].

To simulate the role of AMPK, we included in our model a dependency between mitochondrial health and cellular bioenergetic state, based on the variable environmental energetic stress (E_S_) (Fig 8B). We further included additional rules describing that (I) during bioenergetic stress mitochondrial fission is reduced, resulting in mitochondrial networks which can escape mitophagy [40, 73], and (II) complementation of mitochondrial content by fusion [47] permits *low*-damaged mitochondria to fuse with healthy ones, and recover from the damage (Fig 8C). As such, the probabilities of fusion, fission and biogenesis integrate with the level of bioenergetic stress: a loss of healthy mitochondria intensifies the energy demand level, which, in turn, increases proportionally the probabilities of fusion and biogenesis, and decreases the probability of fission.

Given an initial probability of fusion (P_F0_) and fission (P_f0_) equal to 50% and a probability of biogenesis (P_B0_) equal to 0%, we define the energy stress at time point t, (E_St_), as follows:

$$E_{S}{}_{{}_{t}}=\{\begin{array}{ll} \frac{\left|M_{T_{\left(t-1\right)}}-M_{C}\right|}{M_{C}}, & M_{T_{\left(t-1\right)}}<M_{C}\\ 0, & M_{T_{\left(t-1\right)}}\geq M_{C} \end{array}$$ (6)

Where M_T_ is the total mass of healthy mitochondria and M_C_ is the critical mass (300 μm^2^).

Given a stress level ${\text{E}}_{{\text{S}}_{\text{t}}}$, we defined accordingly the probabilities of fusion (P_F_), fission (P_f_) and biogenesis (P_B_) as:

$$P_{F_{t}}=P_{F_{\left(t-1\right)}}\cdot \left(1+E_{S}{}_{{}_{\left(t-1\right)}}\right)$$ (7)

$$P_{f_{t}}=P_{f_{\left(t-1\right)}}\cdot \left(1-E_{S}{}_{{}_{\left(t-1\right)}}\right)$$ (8)

$$P_{B_{t}}=\{\begin{array}{ll} P_{B_{0}}+E_{S}{}_{{}_{\left(t-1\right)}}\cdot 100, & E_{S}{}_{{}_{\left(t-1\right)}}>0\\ 0, & otherwise \end{array}$$ (9)

Where ${\text{P}}_{{\text{B}}_{0}}$ is the biogenesis probability previously obtained with the optimization, equal to 22.1%. E assumes values in [0,1], while P_F_, P_f_ and P_B_ assume values in [0,100]. The equation governing the biogenesis probability is formulated in order to avoid unrestricted growth, since it links the biogenesis probability to the increase of energetic stress. The receptor threshold was set to 12 minutes, the damage threshold to 12.4 minutes and a degradation frequency to 5.7 minutes (values obtained from the optimization).

A system subjected to continuous damage levels resulted in a loss of healthy mitochondria proportional to the damage intensity. Although the probabilities of fusion, fission and biogenesis varied dynamically with the increased stress, the model was unable to maintain cellular homeostasis (S5 Fig).

However, under normal circumstances, a cell is not constantly subjected to loss of mitochondria; but rather, external factors can induce acute mitochondrial damage, which the cell then eliminates [74–76]. We therefore simulated repeating damage pulses, starting with an initial damage probability of zero, and then transiently (for 1-hour), inducing damage every two hours. Initial probabilities of fusion/fission were set to 50% and biogenesis to 0%. First, we alternated two different probabilities of damage, 10% and 20%, for a period of twelve hours, and subsequently set again the probability of damage to zero (Fig 8D and S6 Movie). During this “*low*-damage” simulation, the mass of damaged mitochondria transiently increased, proportionally to the damage probability, and, after the damage pulses, it was decreasing to zero during the damage-free intervals due to removal by mitophagy (Fig 8E). These dynamics were paralleled by stable variations in the average total healthy mass (Fig 8E), which, in turn, generated small variations in the stress level (Fig 8F). Since the probabilities of fusion, fission and biogenesis are responsive to the stress levels, variations in healthy mass ultimately lead to variations in these probabilities. Thus, under the simulated stress conditions, our model was able to respond to transient damage impulses and maintain the cell close to its initial state. Interestingly, variations were mainly visible in the small-mass mitochondria subpopulation, while the medium and large subpopulations remained nearly unchanged.

We next tested the scenario of increased mitochondrial damage, using with a transient probability of damage of 40%, for one hour every two hours (Fig 8G and S7 Movie). Under these “*high*-damage” conditions, damaged mitochondria accumulated while the mass of healthy mitochondria was subjected to wide variations (Fig 8H), and after twelve hours, the mass of damaged mitochondria was approximately the same as the healthy population. Following the first damage impulse, the dynamic probability rates of fusion and biogenesis were largely increased (Fig 8G), resulting in altered subpopulations. These simulations highlight that biogenesis and degradation are critical limitations to mitochondrial homeostasis, and suggest that experimental focus on the mechanisms which can increase their capacities [7, 77].

The bioenergetic stress response of the two damage scenarios (Fig 8F and 8I) indicate that dynamic adaptation of the probabilities of fusion, fission and biogenesis to the loss of healthy mitochondria is essential in maintain cellular homeostasis. Despite damage-provoked alterations to total mitochondrial mass and subpopulation distributions, the model rapidly regained homeostasis (Fig 8E and 8H). Additionally, since a stress increment is responsible in amplifying the probability of fusion, *low* damaged mitochondria were more subjected to the recovery process (S6B Fig) as highlighted in minor loss in the total healthy mitochondrial mass (S7A Fig) and in the lower level of stress (S7B Fig).

Finally, we assessed parameter influence on this integrated model using sensitivity analysis. We considered eleven parameters, each constrained to the following respective range: MR threshold (0–30), fission probability (0–100), initial mitochondrial mass (50–500), fusion frequency (0–30), fusion probability (0–100), damage probability (0–100), damage threshold (0–30), degradation frequency (0–30), ratio *low/high* damage (0–100), fission frequency (0–30) and biogenesis frequency (0–30). Specifically, we sought to assess how parameter perturbations impact the total mass of healthy mitochondria.

**Fig 9:**
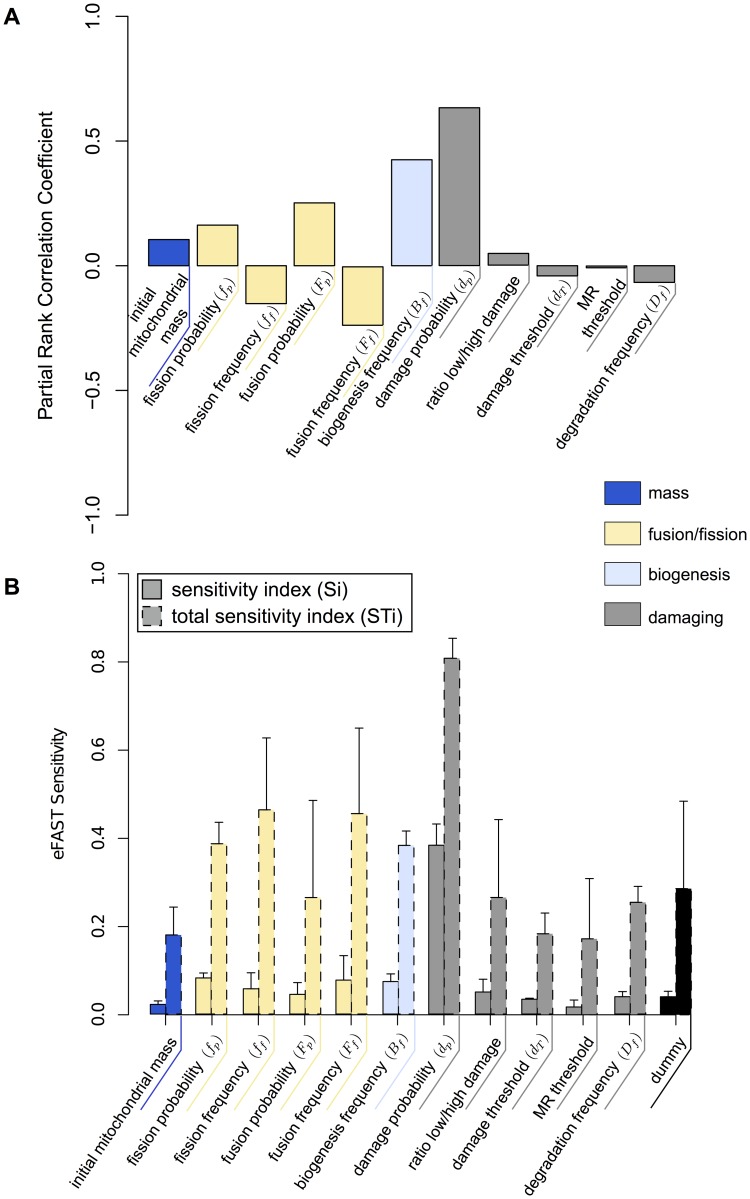
Sensitivity analysis of the full model. (A) Effect of parameters perturbation on the full model. Bar graph represents Partial Rank Correlation Coefficient values for each parameter-output response pairing resulting from a Latin-hypercube sampling of 500 parameter value sets (parameter ranges are described in section “Energetic sensing, mitophagy and mitochondrial biogenesis are integrated processes”). For each parameter set the model was performed over 24 hours. (B) Results of the eFast method used to partition variance in simulation results between parameters. Solid Bars: sensitivity index (Si)–the fraction of output variance explained by the value assigned to that parameter when parameter of interest; Dashed Bars: total sensitivity index (STi)–the variance caused by higher-order non-linear effects between that parameter and the other explored (includes value of Si). Error bars are standard error over four resample curves. Parameter values sets were generated using the sinusoidal sampling approach. 65 parameters were obtained from each of the 4 resampling curves, producing 3120 parameter value sets (260 per parameter).

With the Latin-hypercube sampling approach, five hundred parameter value sets were generated from the parameter space, and a total of 500 simulations (each for the duration of 24 hours) were performed. Taking each parameter in turn, median response values (total mass of healthy mitochondria) were plotted against the parameter value that generated them (S8 Fig), and partial rank correlation coefficients were calculated (Fig 9A). The correlation coefficients, as expected, showed the biogenesis frequency and the damage probability to have significant influence on the simulation response (Fig 9A, S8F and S8G Fig). A high value in the damage probability produces a decrease in the total mass of healthy mitochondria while a high biogenesis frequency reduces the production of new mitochondria. Both of these factors that directly influence the augment in the energy demand of the cell. Moreover, positive correlation in the damage probability occurred mainly for high values of this parameter, greater than ~60%, while low values appeared to be almost irrelevant to the energetic stress (S8G Fig), indicating that the model is able to counter low level of damage. Similarly, the biogenesis frequency has a positive correlation only for high values (S8F Fig), demonstrating that the model is not able to contain energetic stress without an appropriate biogenesis frequency, thus underling the importance of this process in restoring mitochondrial homeostasis during nutrient deprivation. Additionally, similar to the case of transmission dynamic, fission, fusion and degradation frequencies appeared to be negative correlated with the energy demand (S8C, S8E and S8K Fig). While these parameters require small values to rapidly react to the changes, the correlation in these parameters is close to zero, indicating low influence in the energetic stress behavior of the cell.

We further determined parameter impact on model performance. From twelve parameters (eleven plus the ‘dummy’ used for statistical comparison), we took 65 parameter values from each curve, and employed four resampling curves, producing 3120 parameter value sets, 260 per parameter. Plots were created for the energy demand, detailing the first-order (Si) and total-order (STi) sensitivity indexes calculated for each parameter (Fig 9B). The result of the eFAST approach confirmed the precedent sensitivity analysis and indicates an overall general balance among most parameters, with the damage probability to be the most influencing parameter on the model behavior (Fig 9B). Additionally, both two sensitivity analysis methods indicate an overall equilibrium in parameter impact on system behavior, which we interpret to indicate that our final integrative model is not over-parametrized.

## Discussion

The deregulation of mitochondrial dynamics is a contributing factor to many diseases [16, 78, 79]. Here we developed an agent-based model integrating mitochondrial function, dynamics, biogenesis and mitophagy, and investigated how mitochondrial populations establish and maintain homeostasis, and respond to bioenergetic demands. Our modeling approach offers insights into the systemic properties of mitochondrial population dynamics, and sources of cell-to-cell variability.

Under conditions of typical physiological mitochondrial content [49], we predict spatial proximity of mitochondria to be a major determinant for distribution of mitochondrial morphologies. Since mitochondria must interact in order to fuse, mitochondrial density contributes independently of a probability for fusion and fission. Importantly, variations in the total mass of mitochondria affect both heterogeneity of the intracellular mitochondria population and therefore resulted in cell-to-cell variability. We found that higher mitochondrial masses are more responsive to the probability of fusion, and in this case physical proximity does not represent a limiting factor. As a result, higher mitochondrial masses allow for more heterogeneous populations and lead to less cell-to-cell variability. Further, our simulations of network connectivity revealed that fusion and fission probabilities have a minor impact compared to velocities which determine the mobility of mitochondria. Importantly, when integrating isolated mitochondrial biogenesis and degradation processes, we did not obtain stable behavior in mitochondrial morphology dynamics and mass. Only by implementing crosstalk between cellular bioenergetic state and mitochondrial morphologies were we able to obtain a homeostatic-capable dynamic model.

Thus, our integrative approach suggests that model-based investigation of organelle dynamics requires incorporating functional and environmental considerations. Similarly, we previously found that in order to accurately simulate experimental data, modeling autophagy dynamics required the consideration of bi-directional signaling, in which recycled nutrients, provided through autophagy activation, establish a negative feedback on autophagy [27]. Here, our modeling approach highlights the limitations of mitochondrial mass homeostasis. Most obviously, during prolonged stress mitochondrial biogenesis is not sufficient to compensate in response to increasing mitochondrial stress, and mitophagy. These limitations directly relate to the role of mitochondria in cell death signaling, and future work will focus on integrating apoptotic stress and different modes of regulated mitophagy [7, 80].

An important advantage of our approach to multi-scale modeling is that parameters can be used to account for qualitatively- and quantitatively-measured behavior. Here we employed parameter sweeping [63, 81] to find parameter combinations which produced the best description of experimentally-established dynamics of mitochondrial growth and degradation, and two sensitivity analysis methods [57, 82] to analyze the influence of the parameters on transmission dynamic and energetic sensing. Parameter sweeping identified parameters that best predict experimental observations. For example, optimizing mitophagy, our model predicts recognition and sequestration dynamics (a receptor threshold of 12 minutes, and a damage threshold of 12.4 minutes) and a subsequent degradation event of 5.7 minutes. Thereby these values represent a precise description of cellular mechanisms to be experimentally tested, which has recently become possible due to advances in biosensors for measuring mitochondrial damage, mitophagy and biogenesis (reviewed in [8]). Further, by analyzing the distributions of the parameters obtained from the optimization, and their influence by the sensitivity analysis, we gain insight into parameter impact on model behavior, and relationships between parameters. The result of two different global sensitivity analysis methods on our model in the case of transmission dynamic confirmed our hypothesis that mitochondrial mobility is an essential component for transmission dynamics rather than the total mitochondrial mass. Similarly, in the context of energetic sensing, the model appeared to be mainly subjected to the damage probability and to the frequencies of fusion, fission and biogenesis, determinant components for maintaining homeostasis in the cell. Further, low correlation and an overall equilibrium between parameters suggest that our model performs robustly and is not over-parameterized. This modeling approach could be adopted as framework to transform experimental observation into quantitative statements, thereby driving follow-up target experiments. However, it is important to note that our model represents a generalized mammalian cell. As mitochondrial dynamics and morphologies, as well as cellular size and architecture, vary among cell types [3, 83, 84], our model can be adapted for cell-type specific simulations. For example, altering cellular dimensions in order to determine the impact of restricted mitochondrial movements in neuronal processes [33, 37], or restricting mitochondrial movement in order to analyze the highly-static and ordered mitochondrial arrangement in cardiomyocytes [85].

In summary, we propose that ABM is uniquely suited to model complex, multi-scale systems such as mitochondrial dynamics and morphology, and advances model-guided investigation of sources of subcellular heterogeneities towards the goal of understanding cellular behavior, and emergence of cell heterogeneity.

## Materials and Methods

### Modeling

ABM was implemented using the multi-agent programmable modeling environment NetLogo (v5.2.0) [30]. In NetLogo, mobile agents (*turtles*) interact in a 2-dimensional environment subdivided into micro-compartments (*patches*). Our model consists of five classes of agents, two static (cell and nucleus) and three dynamic (mitochondria, autophagososmes and autolysosomes), in particular:

- cell (static agent): it contains all the other agents, represents the external boundary of the system beyond which mobile agents are not allowed to exceed,
- nucleus (static agent): it represents the internal boundary of the system inside which mobile agents are not allowed to enter,
- mitochondria (mobile agents): they are able to move inside the cell assuming variable velocity (v_l_ and v_h_ depending on their position in the cell), rotate in relation to an angle (ϑ_1_ and ϑ_2_ depending on their surrounding) and with the properties to undergo fusions (according to a fusion probability F_p_ and at a fusion frequency F_f_), fission (according to a fission probability f_p_ and at a fission frequency f_f_) and biogenesis (according to a biogenesis probability B_p_ and at a biogenesis frequency B_f_). Also, they can undertake a damage state (according to a damage probability D_p_),
- autophagosomes (mobile agents): they are able to move inside the cell and they are involved in the mitochondrial degradation process representing sequestering autophagosomes,
- autolysosomes (mobile agents): they are able to move inside the cell and they are involved in the mitochondrial degradation process representing degrading lysosomes,

a more detailed description of the model agents is available in the Result section.

In order to map the time and space of our model to real physical time and space we decided that a distance of 1 in our simulation space corresponds to a physical distance of 1 μm and each time step of our simulation corresponds to 1 seconds. Therfore, dt and ds are defined as follows:

$$dt=\;\frac{1\;sec.}{1\;time\;step}$$

$$ds=\;\frac{1\;\mu m}{1\;patch}$$

Accordingly, in order to convert the real velocity to a NetLogo velocity v_NL_, we defined:

$$v_{NL}=v\cdot \frac{dt}{ds},$$

where v assumes the values v_l_ or v_h_ previously defined in the text.

As a conceptual characterization of the ABM approach adopted in this work we present here a detailed explanation of the model iterations describing the mitochondrial fusion and fission cycle (Fig 1B and 1C and S1 Fig). At time point dt = 0 the model is initialized and mitochondria are assigned a random position inside the cell with and a random size (ranging continuously from M_min_ to M_max_) until a total initial mass value, decided from the user (e.g. 300 μm^2^), is reached. In the first time step (dt = 1) each mitochondrion first moves and rotates (as explained in the results section) then the fusion and fission cycle starts. The program loops over all mitochondria, starting from a randomly chosen mitochondrion *j*, if the time step matches the fusion frequency (F_f_) (e.g. if it is equal to 5 minutes or a multiple of 5 minutes) then a random number is sampled uniformly between 0 and 100, if this number is less than the fusion probability (F_p_) the program checks the distance to the closest mitochondrion, if (I) the distance is less or equal to the dimension of *j*, the program controls if (II) the mass of *j* plus the mass of the closest mitochondrion is less or equal to M_max_, if this conditions are satisfied the fusion process occurs (if either one of these two conditions are not satisfied the program switch to the next mitochondrion). The two mitochondria fuse to a resulting mitochondrion with a mass equal to the sum of the two masses. The model cycles over all the mitochondria repeating the procedures described above and then moves to the fission process. In the fission process the program loops again over all mitochondria. Starting from a randomly chosen mitochondrion *j*, if the time step matches the fission frequency (f_f_) (e.g. if it is equal to 5 minutes or a multiple of 5 minutes) then a random number is sampled uniformly between 0 and 100, if this number is less than the fission probability (f_p_), the program checks if the mass of *j* is greater or equal to 2M_min_. If this condition is satisfied the fission process occurs (if not the program switch to the next mitochondrion). The mitochondrion *j* splits then in two mitochondria of size M_1_ and M_2_ given by Eqs (1) and (2). The model cycles over all the mitochondria repeating the procedures described above. Following, the time is incremented of one unit (t = t + Δt) and all the process is repeated until reaching the final time point desired (chosen by the user).

The fusion and fission cycle was taken as example since it is both the simplest and the core of the program. The model increases then in complexity and new processes are integrated (S4 and S6 Figs) nevertheless, they work along the lines of this basic procedure.

### Optimization

Optimization search was performed with the R package for genetic algorithm [63] using a classic Genetic Algorithm (settings: type = real-valued, popSize = 10, maxfitness = 0, pcrossover = 0.8, pmutation = 0.01). In order to optimize the two objective functions O_B_ and O_D_ (defined in the text) we performed each time 100 iteration. Moreover, in the optimization process, the objective function analyzed at each time step is an average of 10 model simulation runs. The parameters optimized (biogenesis probability, biogenesis frequency, damage threshold, receptor threshold and degradation frequency) were constrained in the range [1,60].

### Sensitivity analysis

Sensitivity analysis was performed with the Simulation Parameter Analysis R Toolkit ApplicatioN: Spartan (v2.2.1) [57, 82, 86], specifically using the Latin-Hypercube Sampling and Analysis and the eFast Sampling and Analysis. In the section “Transmission dynamics of the mitochondrial network” the GFP-labeled mass of mitochondria was used as a response variable, while in the section “Energetic sensing, mitophagy and mitochondrial biogenesis must be considered as integrated processes” the total mass of healthy mitochondria was used as a response variable.

### Additional analysis

Statistical analysis and plotting were executed with the open source environment for statistical computing R (v3.2.1) [87]. All the simulations were performed in R profiting of the link between R and NetLogo given by the R package RNetLogo (v1.0–1) [88, 89].

## Supporting Information

S1 Fig Fusion/fission cycle algorithm. Detailed description of the complete algorithm for the fusion/fission cycle represented in Fig 1D.
(TIF)

S2 Fig Compound effects between parameters using Latin-hypercube parameter sampling. (A)Fusion frequency sorted by the value assigned to total mass.(B)Fusion frequency sorted by the value assigned to initial labeled mass.(C)Fusion frequency sorted by the value assigned to fission probability.(D)Fusion frequency sorted by the value assigned to fission frequency.(E)Fusion frequency sorted by the value assigned to fusion probability.(F)Fusion frequency sorted by the value assigned to fusion frequency.(G)Fusion frequency sorted by the value assigned to v_l_.(H)Fusion frequency sorted by the value assigned to v_h_.
Red lines represent the *lowess* smoother lines.
(TIFF)

S3 Fig Subpopulation analysis with and without integrated cellular bioenergetics state. (A)Line graphs display mean value and standard deviation (shaded regions) of 100 simulations of total mitochondria mass for an initial mass of 150 fragmented mitochondria with (dark grey line) and without (light grey line) biogenesis integrated with cellular bioenergetics state. Initial parameters values: fusion probability = fission probability = 50%, fusion frequency = fission frequency = 5 minutes, biogenesis probability = 22.1%, biogenesis frequency = 28.9 minutes, receptor threshold = 12 minutes, damage threshold = 12.4 minutes, degradation frequency = 5.7 minutes). All simulations were performed for a total time of 12 hours.(B)Line graphs display mean value and standard deviation (shaded regions) of 100 simulations of total mitochondria mass for an initial mass of 150 fragmented mitochondria and with biogenesis integrated with cellular bioenergetics state subjected to three different initial biogenesis frequency values: 28.9 (light blue line), 57.8 (blue line) and 86.7 (dark blue line) minutes. Initial parameters values: fusion probability = fission probability = 50%, fusion frequency = fission frequenc*y* = 5 minutes, receptor threshold = 12 minutes, damage threshold = 12.4 minutes, degradation frequency = 5.7 minutes). All simulations were performed for a total time of 12 hours.Line graphs display mean value and standard deviation (shaded regions) of 100 simulations for an initial total mitochondrial mass of 150 fragmented mitochondria of the total mitochondrial mass (grey line) and three mitochondrial subpopulations: small mass (orange line), medium mass (blue line) and large mass (green line) without biogenesis integrated with cellular bioenergetics state. Initial parameters values: fusion probability = fission probability = 50%, fusion frequency = fission frequency = 5 minutes, biogenesis probability = 22.1%, biogenesis frequency = 28.9 minutes, receptor threshold = 12 minutes, damage threshold = 12.4 minutes, degradation frequency = 5.7 minutes). All simulations were performed for a total time of 12 hours.Same as (C) but with biogenesis integrated with cellular bioenergetics state.
(TIFF)

S4 Fig Degradation cycle algorithm. Detailed description of the complete algorithm for the degradation cycle represented in Fig 7B.
(TIFF)

S5 Fig (A)Line graphs display mean value and standard deviation (shaded regions) of 100 simulations of probabilities of fusion (dark green line), fission (light green line), biogenesis (orange line) total mitochondrial mass (grey line), three mitochondria subpopulations: small mass (orange line), medium mass (blue line) and big mass (green line), and bioenergetics stress (red line) subjected to a constant damage signals of 5%. The plot represents the evolution of the total mass normalized to the total mass at time point 0. Initial values assigned to the model: fusion probability = fission probability = 50%, fusion frequency = fission frequency = 5 minutes, biogenesis probability = 22.1%, biogenesis frequency = 28.9 minutes, receptor threshold = 12 minutes, damage threshold = 12.4 minutes, degradation frequency = 5.7 minutes. All the simulations were performed for a total time of 24 hours.(B)Same as (A) but with constant damage signals of 20%.(C)Same as (A) but with constant damage signals of 40%.(D)Same as (A) but with constant damage signals of 60%.
(TIFF)

S6 Fig Recovery algorithm. Detailed description of the complete algorithm for the recovery cycle.
(TIFF)

S7 Fig Energetic sensing and recovery. (A)Line graphs display mean value and standard deviation (shaded area) of 100 simulations for an initial mitochondrial mass of 300 of the total mitochondrial mass with (dark grey) and without (light grey) the recovery process, subjected to five different damage signals (40%) every two hours for one hour with and without recovery process. The plot represents the evolution of the total mass and of three mitochondrial subpopulations normalized to the total mass at time point 0. Initial parameters values: fusion probability = fission probability = 50%, fusion frequency = fission frequency = 5 minutes, biogenesis probability = 22.1%, biogenesis frequency = 28.9 minutes, receptor threshold = 12 minutes, damage threshold = 12.4 minutes, degradation frequency = 5.7 minutes). Line graphs represent the mean value of 100 simulations. All the simulations were performed for a total time of 24 hours.(B)Line graphs display mean value and standard deviation (shaded area) of 100 simulations for an initial mitochondrial mass of 300 of the bioenergetics stress with (dark red) and without (light red) the recovery process, subjected to five different damage signals (40%) every two hours for one hour with and without recovery process. The plot represents the evolution of the total mass and of three mitochondrial subpopulations normalized to the total mass at time point 0. Initial parameters values: fusion probability = fission probability = 50%, fusion frequency = fission frequency = 5 minutes, biogenesis probability = 22.1%, biogenesis frequency = 28.9 minutes, receptor threshold = 12 minutes, damage threshold = 12.4 minutes, degradation frequency = 5.7 minutes). Line graphs represent the mean value of 100 simulations. All the simulations were performed for a total time of 24 hours.
(TIFF)

S8 Fig Compound effects between parameters using Latin-hypercube parameter sampling. (A)MR threshold sorted by the value assigned to total mass.(B)MR threshold sorted by the value assigned to fission probability.(C)MR threshold sorted by the value assigned to fission frequency.(D)MR threshold sorted by the value assigned to fusion probability.(E)MR threshold sorted by the value assigned to fusion frequency.(F)MR threshold sorted by the value assigned to biogenesis frequency.(G)MR threshold sorted by the value assigned to EN stress level.(H)MR threshold sorted by the value assigned to ration L/H damage.(I)MR threshold sorted by the value assigned to damage threshold.(J)MR threshold sorted by the value assigned to MR threshold.(K)MR threshold sorted by the value assigned to degradation frequency.
Red lines represent the *lowess* smoother lines.
(TIFF)

S1 Code Mitochondrial population model. NetLogo File for the mitochondrial population full model including fusion/fission, biogenesis, damage and degradation cycles and energetic sensing. Load and use with NetLogo 5.2.0 or newer.
(NLOGO)

S1 Movie Time course movie of central mitochondrial dynamic: fusion-fission cycles. This movie shows the time-course of one model run describing mitochondrial directionality. Starting with an initial total mass of 300 mitochondria, the movie represents the evolution of the population with fusion/fission probabilities of 50%/50% and fusion/fission frequencies of 5 minutes (Fig 2C middle graph).
(AVI)

S2 Movie Time course movie of mitochondrial directionality. This movie shows the time-course of one model run describing mitochondrial directionality. Starting with an initial total mass of 300 mitochondria, the movie represents the evolution of the population with fusion/fission probabilities of 50%/50%, fusion/fission frequencies of 5 minutes and ϑ_1_ ϵ [0°, 10°] (Fig 3B).
(AVI)

S3 Movie Time course movie of transmission dynamic in mitochondrial network. This movie shows the time-course of one model run describing transmission dynamic in mitochondrial network. Starting with an initial total mass of 300 mitochondria with 10% of them GFP labeled (green), the movie represents the GFP transmission through the cell. Fusion/fission probabilities of 50%/50% and fusion/fission frequencies of 5 minutes (Fig 4B lower graph).
(AVI)

S4 Movie Time course movie of mitochondrial biogenesis. This movie shows the time-course of one model run describing mitochondrial biogenesis. Starting with an initial mitochondrial mass of 1, the movie represents the repopulation of the cell. Fusion/fission probabilities of 50%/50%, fusion/fission frequencies of 5 minutes, biogenesis probability of 22.1% and biogenesis frequency of 28.9 minutes (Fig 6D).
(AVI)

S5 Movie Time course movie of mitochondrial damage sensing and mitophagy. This movie shows the time-course of one model run describing mitochondrial damage sensing and mitophagy. Starting with an initial mitochondrial mass of 300 (50% high-damaged and 50% low-damaged), the movie represents mitochondrial degradation (Fig 7E). Receptor threshold of 12 minutes, damage threshold of 12.4 minutes and degradation frequency of 5.7 minutes.
(AVI)

S6 Movie Time course movie of mitochondrial energetic sensing (low stress). This movie shows the time-course of one model run describing mitochondrial energetic sensing. Starting with an initial mitochondrial mass of 300 subjected to five different damage signals (10% and 20%) every two hours for one hour, the movie represents the evolution of the population (Fig 8E).
(AVI)

S7 Movie Time course movie of mitochondrial energetic sensing (high stress). This movie shows the time-course of one model run describing mitochondrial energetic sensing. Starting with an initial mitochondrial mass of 300 subjected to five different damage signals (40%) every two hours for one hour, the movie represents the evolution of the population (Fig 8H).
(AVI)
