# Supplementary figures and images for: Agent-Based Modeling of Mitochondria Links Sub-Cellular Dynamics to Cellular Homeostasis and Heterogeneity
Source: PLoS One. 2017 Jan 6;12(1):e0168198. doi: 10.1371/journal.pone.0168198 (PMC5217980; doi:10.1371/journal.pone.0168198)

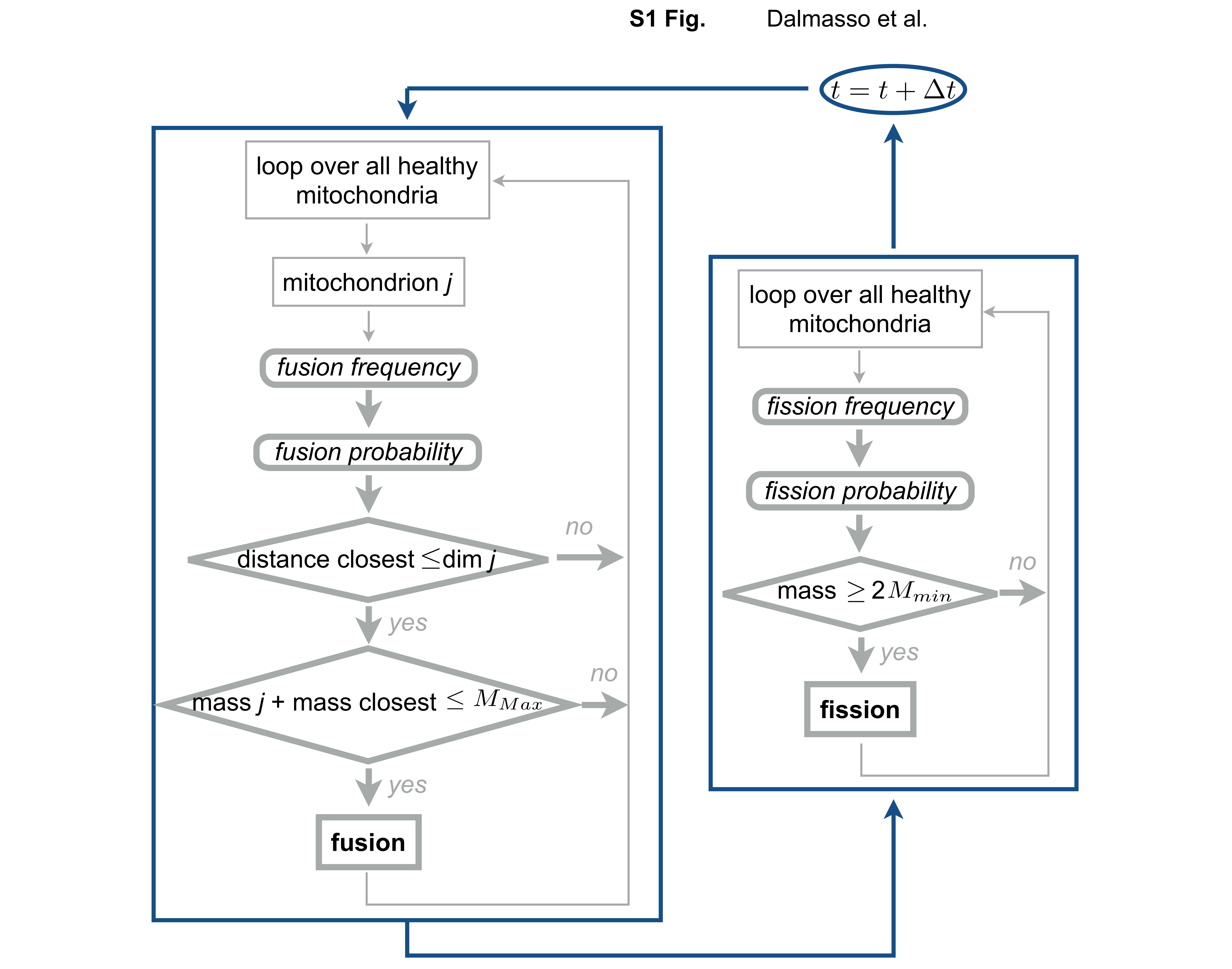

Supplement: S1 Fig — Detailed description of the complete algorithm for the fusion/fission cycle represented in Fig 1D. (TIF) [file pone.0168198.s001.tif]

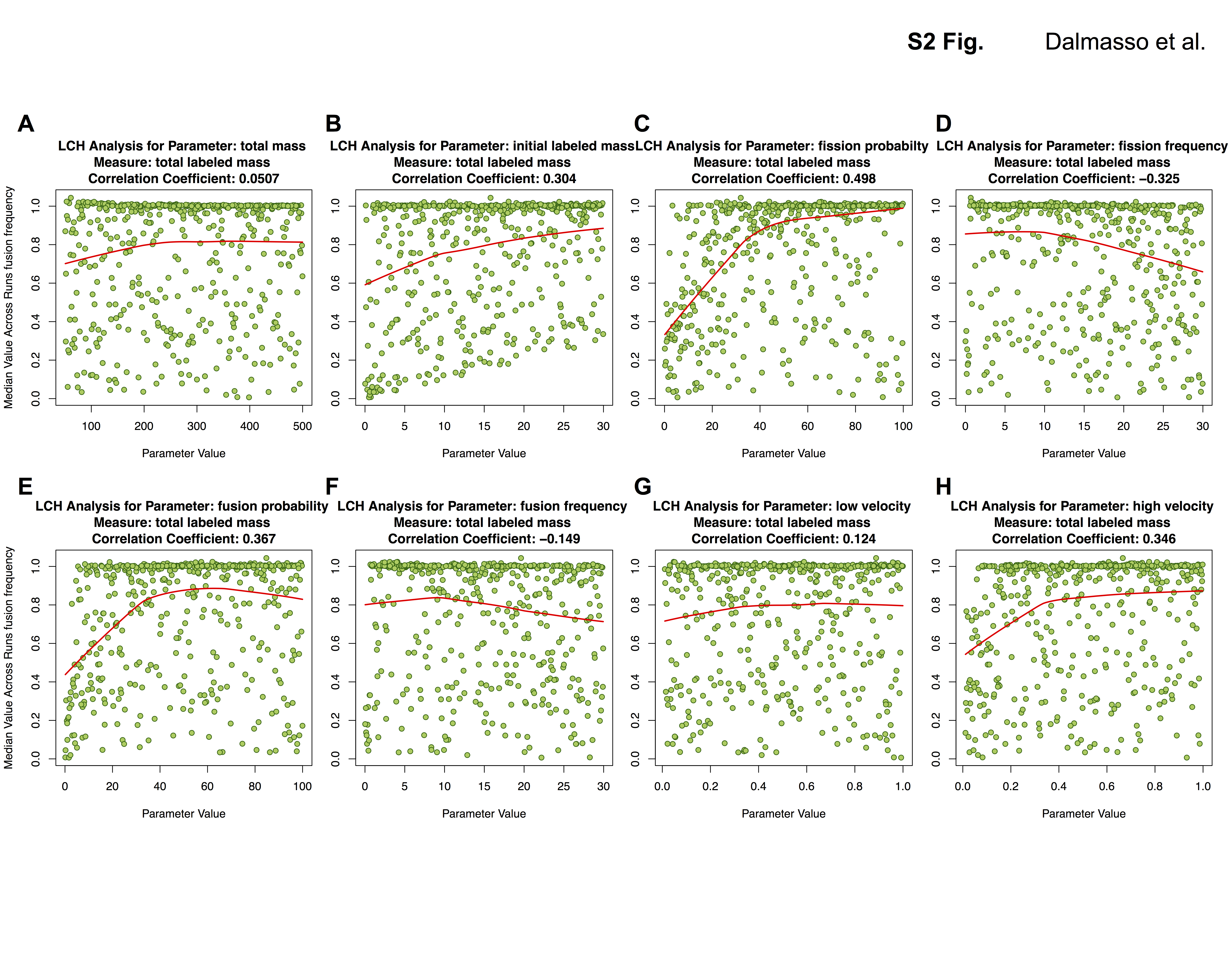

Supplement: S2 Fig — (A)Fusion frequency sorted by the value assigned to total mass.(B)Fusion frequency sorted by the value assigned to initial labeled mass.(C)Fusion frequency sorted by the value assigned to fission probability.(D)Fusion frequency sorted by the value assigned to fission frequency.(E)Fusion frequency sorted by the value assigned to fusion probability.(F)Fusion frequency sorted by the value assigned to fusion frequency.(G)Fusion frequency sorted by the value assigned to vl.(H)Fusion frequency sorted by the value assigned to vh. Red lines represent the lowess smoother lines. (TIFF) [file pone.0168198.s002.tiff]

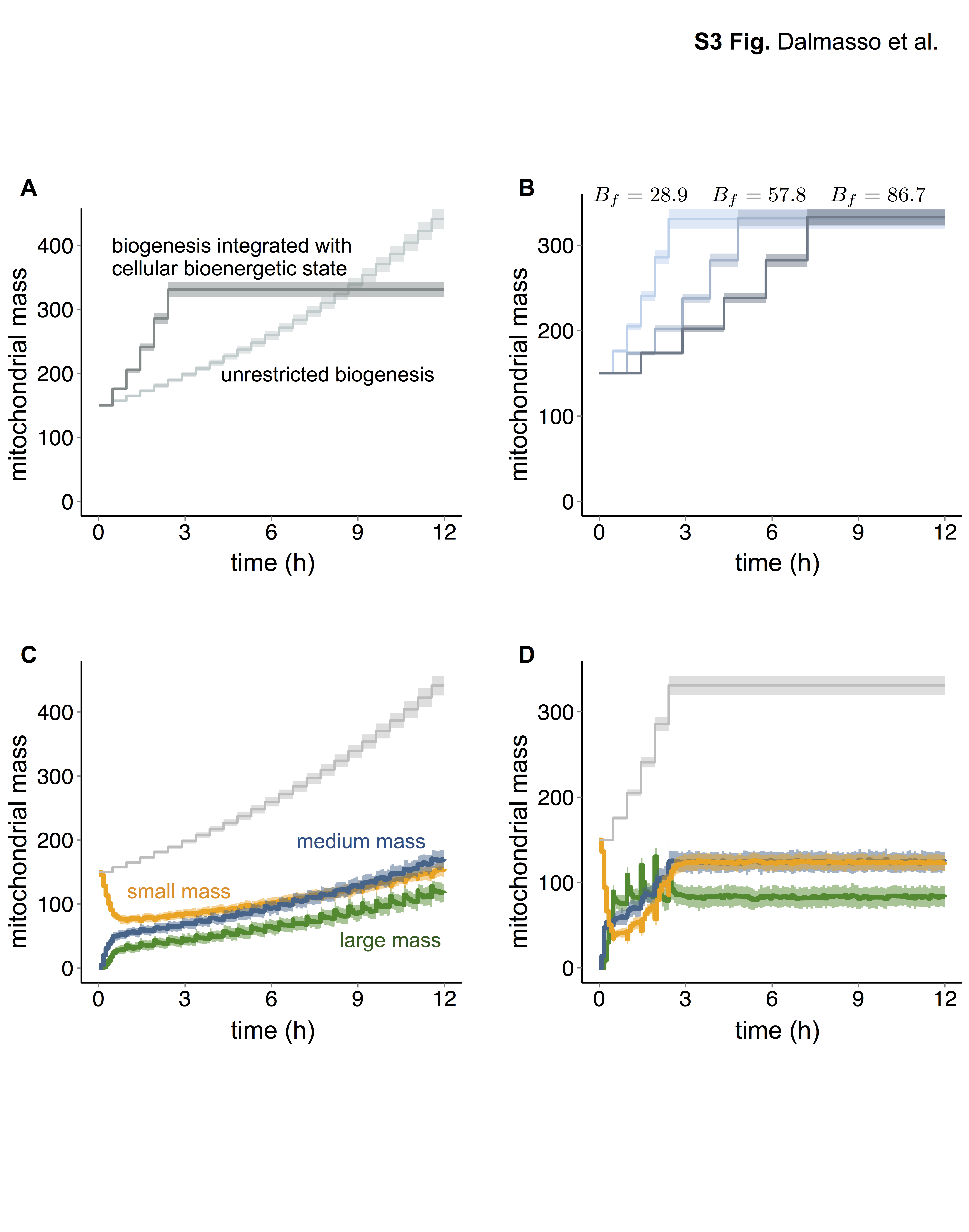

Supplement: S3 Fig — (A)Line graphs display mean value and standard deviation (shaded regions) of 100 simulations of total mitochondria mass for an initial mass of 150 fragmented mitochondria with (dark grey line) and without (light grey line) biogenesis integrated with cellular bioenergetics state. Initial parameters values: fusion probability = fission probability = 50%, fusion frequency = fission frequency = 5 minutes, biogenesis probability = 22.1%, biogenesis frequency = 28.9 minutes, receptor threshold = 12 minutes, damage threshold = 12.4 minutes, degradation frequency = 5.7 minutes). All simulations were performed for a total time of 12 hours.(B)Line graphs display mean value and standard deviation (shaded regions) of 100 simulations of total mitochondria mass for an initial mass of 150 fragmented mitochondria and with biogenesis integrated with cellular bioenergetics state subjected to three different initial biogenesis frequency values: 28.9 (light blue line), 57.8 (blue line) and 86.7 (dark blue line) minutes. Initial parameters values: fusion probability = fission probability = 50%, fusion frequency = fission frequency = 5 minutes, receptor threshold = 12 minutes, damage threshold = 12.4 minutes, degradation frequency = 5.7 minutes). All simulations were performed for a total time of 12 hours.Line graphs display mean value and standard deviation (shaded regions) of 100 simulations for an initial total mitochondrial mass of 150 fragmented mitochondria of the total mitochondrial mass (grey line) and three mitochondrial subpopulations: small mass (orange line), medium mass (blue line) and large mass (green line) without biogenesis integrated with cellular bioenergetics state. Initial parameters values: fusion probability = fission probability = 50%, fusion frequency = fission frequency = 5 minutes, biogenesis probability = 22.1%, biogenesis frequency = 28.9 minutes, receptor threshold = 12 minutes, damage threshold = 12.4 minutes, degradation frequency = 5.7 minutes). All simul [file pone.0168198.s003.tiff]

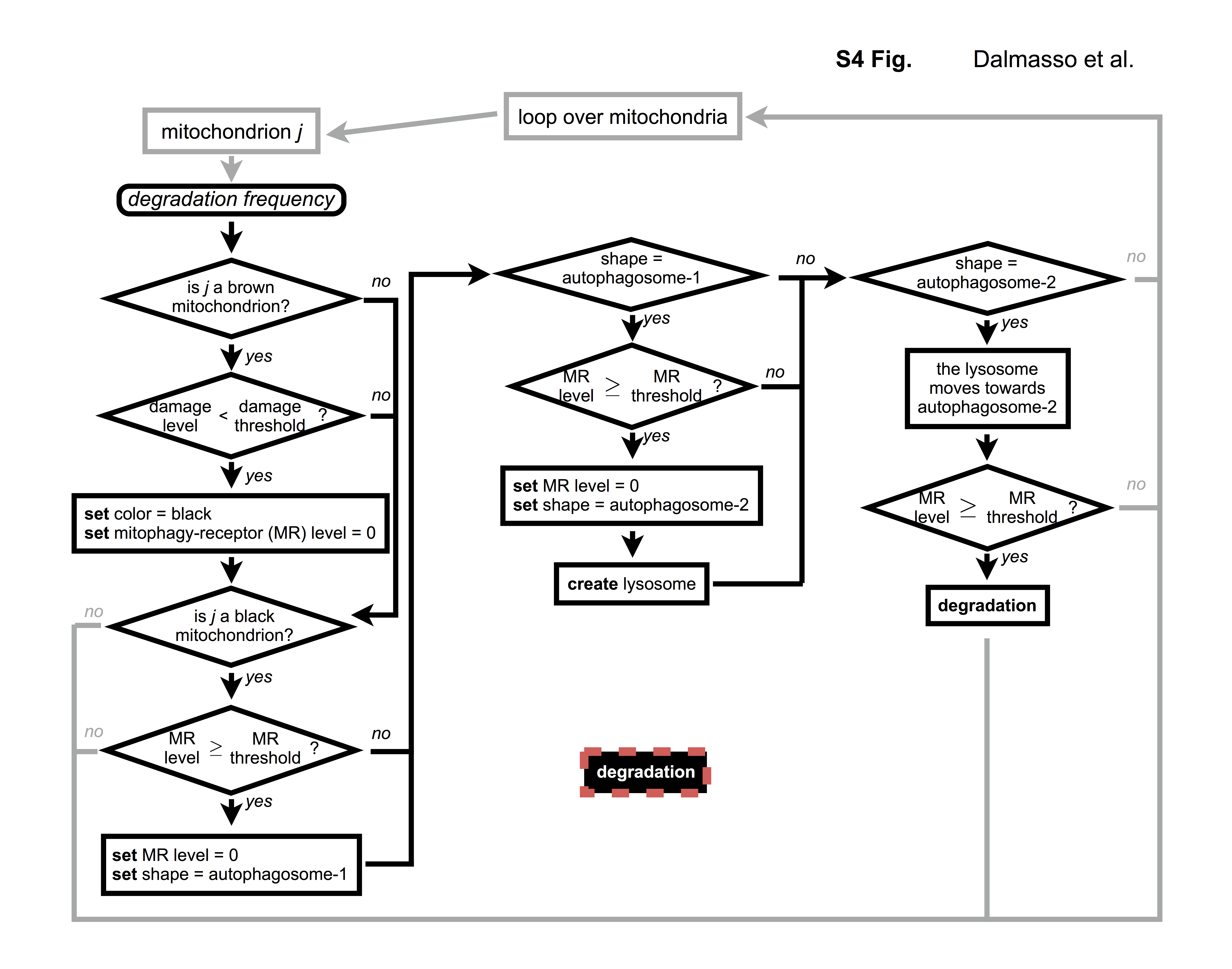

Supplement: S4 Fig — Detailed description of the complete algorithm for the degradation cycle represented in Fig 7B. (TIFF) [file pone.0168198.s004.tiff]

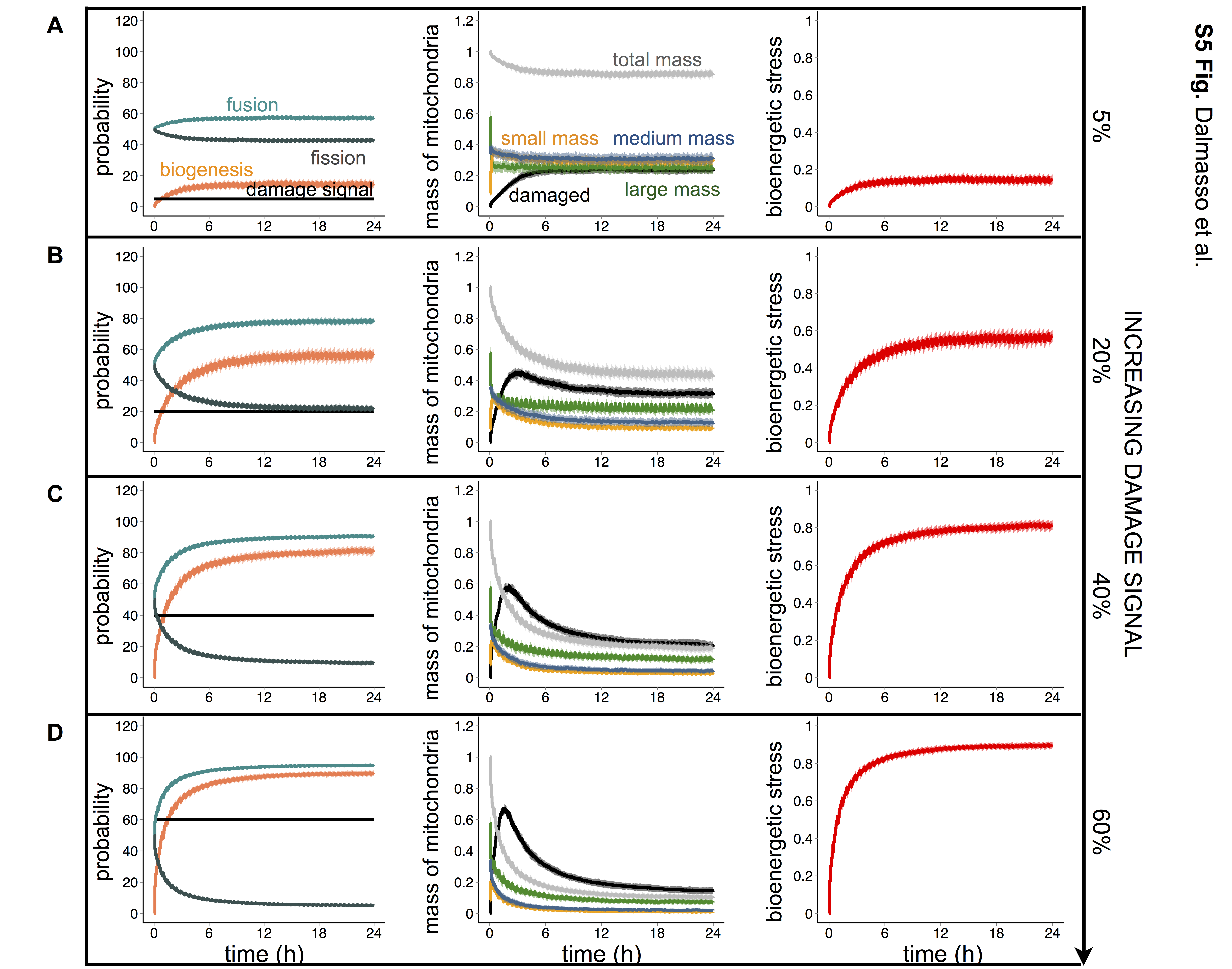

Supplement: S5 Fig — (A)Line graphs display mean value and standard deviation (shaded regions) of 100 simulations of probabilities of fusion (dark green line), fission (light green line), biogenesis (orange line) total mitochondrial mass (grey line), three mitochondria subpopulations: small mass (orange line), medium mass (blue line) and big mass (green line), and bioenergetics stress (red line) subjected to a constant damage signals of 5%. The plot represents the evolution of the total mass normalized to the total mass at time point 0. Initial values assigned to the model: fusion probability = fission probability = 50%, fusion frequency = fission frequency = 5 minutes, biogenesis probability = 22.1%, biogenesis frequency = 28.9 minutes, receptor threshold = 12 minutes, damage threshold = 12.4 minutes, degradation frequency = 5.7 minutes. All the simulations were performed for a total time of 24 hours.(B)Same as (A) but with constant damage signals of 20%.(C)Same as (A) but with constant damage signals of 40%.(D)Same as (A) but with constant damage signals of 60%. (TIFF) [file pone.0168198.s005.tiff]

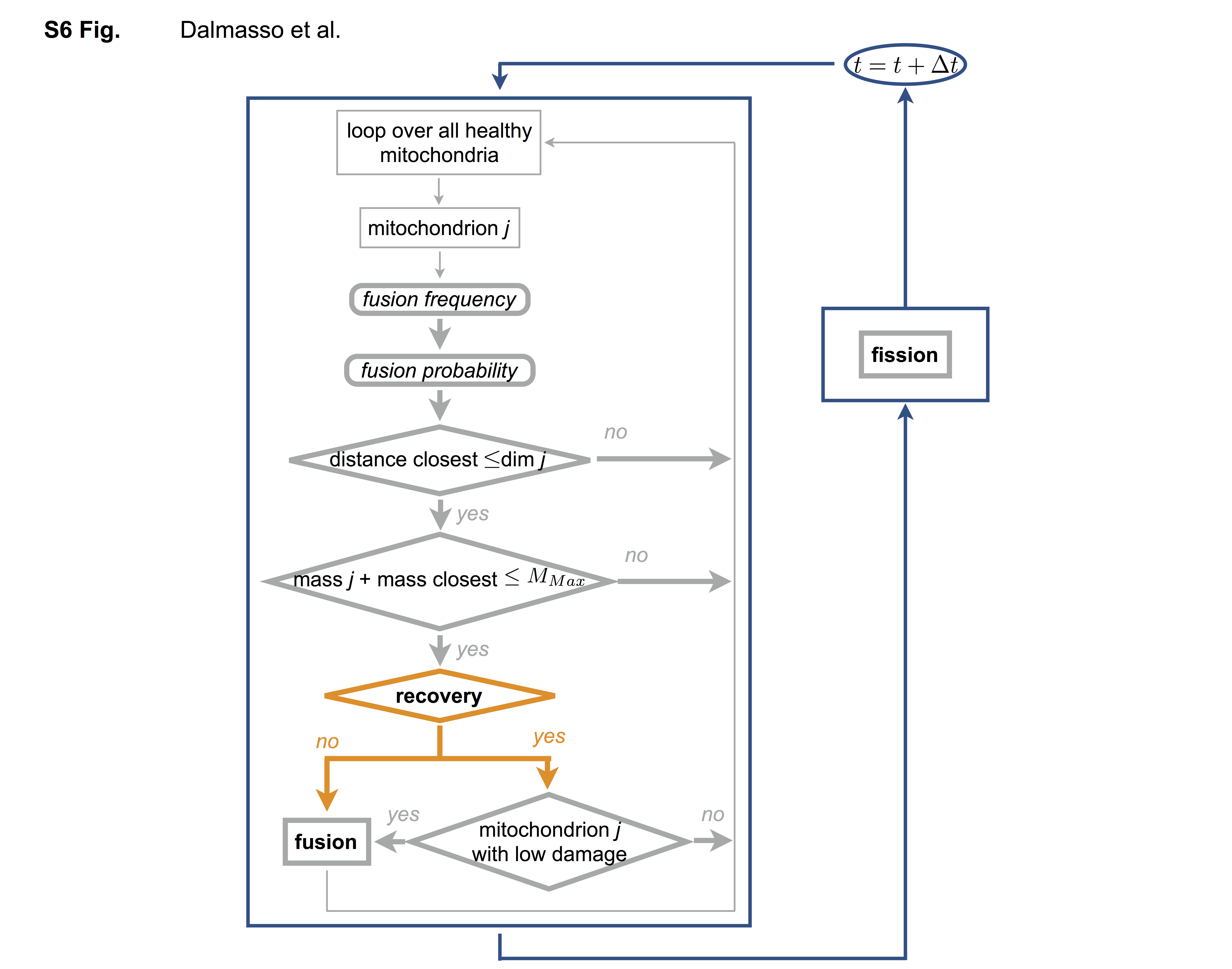

Supplement: S6 Fig — Detailed description of the complete algorithm for the recovery cycle. (TIFF) [file pone.0168198.s006.tiff]

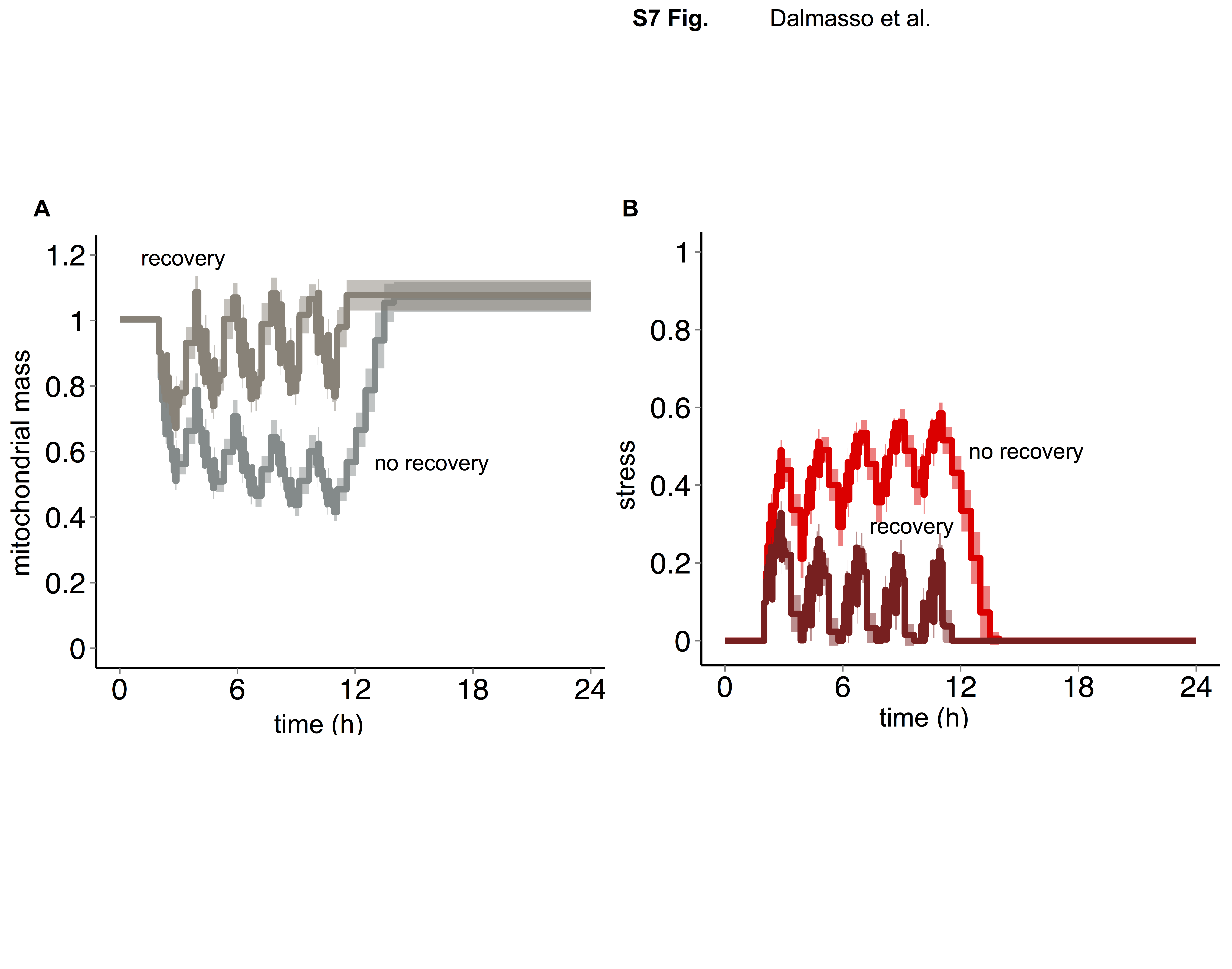

Supplement: S7 Fig — (A)Line graphs display mean value and standard deviation (shaded area) of 100 simulations for an initial mitochondrial mass of 300 of the total mitochondrial mass with (dark grey) and without (light grey) the recovery process, subjected to five different damage signals (40%) every two hours for one hour with and without recovery process. The plot represents the evolution of the total mass and of three mitochondrial subpopulations normalized to the total mass at time point 0. Initial parameters values: fusion probability = fission probability = 50%, fusion frequency = fission frequency = 5 minutes, biogenesis probability = 22.1%, biogenesis frequency = 28.9 minutes, receptor threshold = 12 minutes, damage threshold = 12.4 minutes, degradation frequency = 5.7 minutes). Line graphs represent the mean value of 100 simulations. All the simulations were performed for a total time of 24 hours.(B)Line graphs display mean value and standard deviation (shaded area) of 100 simulations for an initial mitochondrial mass of 300 of the bioenergetics stress with (dark red) and without (light red) the recovery process, subjected to five different damage signals (40%) every two hours for one hour with and without recovery process. The plot represents the evolution of the total mass and of three mitochondrial subpopulations normalized to the total mass at time point 0. Initial parameters values: fusion probability = fission probability = 50%, fusion frequency = fission frequency = 5 minutes, biogenesis probability = 22.1%, biogenesis frequency = 28.9 minutes, receptor threshold = 12 minutes, damage threshold = 12.4 minutes, degradation frequency = 5.7 minutes). Line graphs represent the mean value of 100 simulations. All the simulations were performed for a total time of 24 hours. (TIFF) [file pone.0168198.s007.tiff]

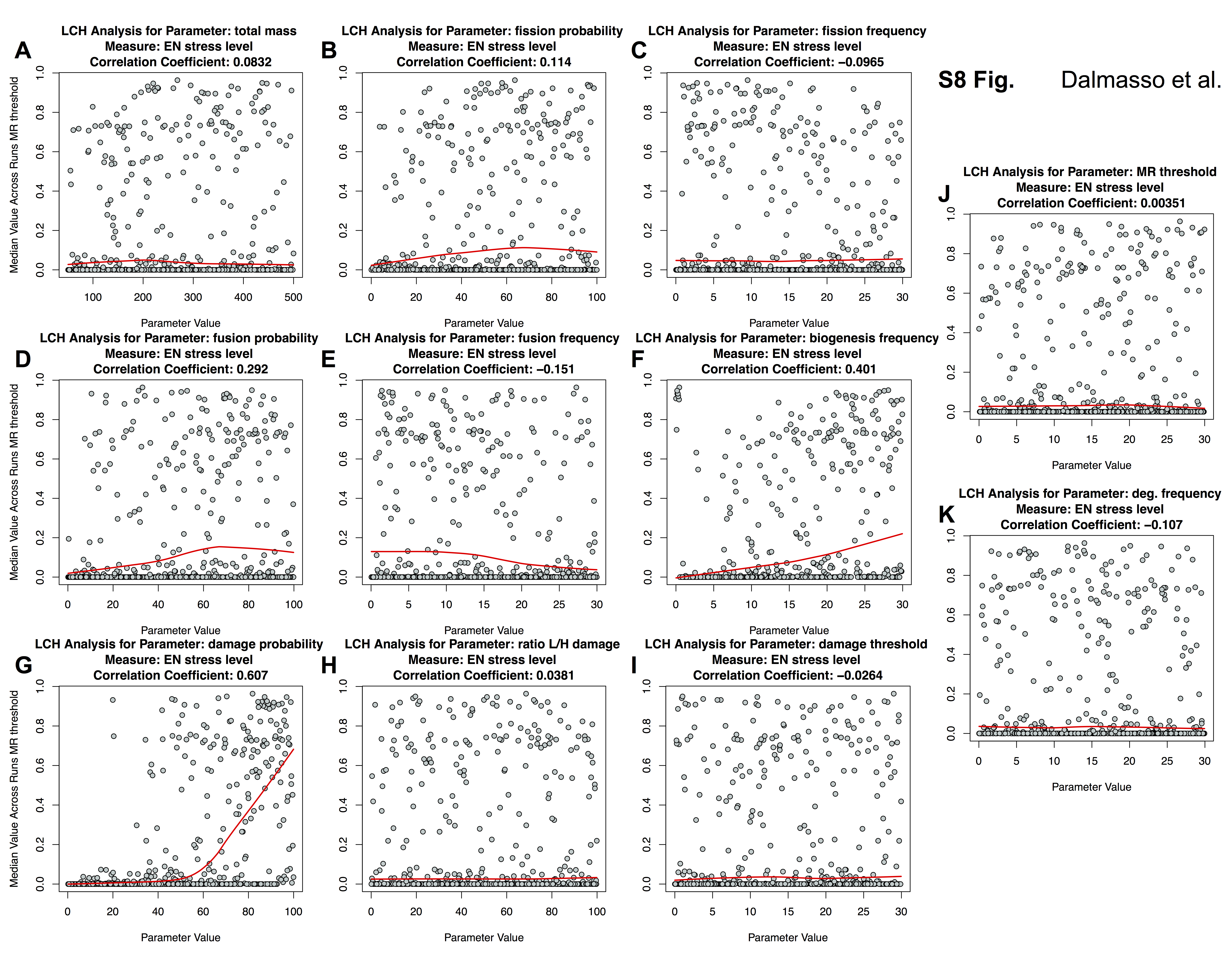

Supplement: S8 Fig — (A)MR threshold sorted by the value assigned to total mass.(B)MR threshold sorted by the value assigned to fission probability.(C)MR threshold sorted by the value assigned to fission frequency.(D)MR threshold sorted by the value assigned to fusion probability.(E)MR threshold sorted by the value assigned to fusion frequency.(F)MR threshold sorted by the value assigned to biogenesis frequency.(G)MR threshold sorted by the value assigned to EN stress level.(H)MR threshold sorted by the value assigned to ration L/H damage.(I)MR threshold sorted by the value assigned to damage threshold.(J)MR threshold sorted by the value assigned to MR threshold.(K)MR threshold sorted by the value assigned to degradation frequency. Red lines represent the lowess smoother lines. (TIFF) [file pone.0168198.s008.tiff]
